# Thermally Tunable Fading Memory Enabled by Opto-Ionic-Electronic Coupling in AgInP_2_S_6_‑Gated Two-Dimensional Field-Effect Transistors

**DOI:** 10.1021/acsnano.5c21149

**Published:** 2026-10-05

**Authors:** Dipanjan Sen, Anirban Chowdhury, Anshul Rasyotra, Lei Ding, Rui Gusmao, Subir Ghosh, Arpan Ghosh, Joan M. Redwing, Divya Somvanshi, Yang Yang, Zdenek Sofer, Saptarshi Das

**Affiliations:** † Engineering Science and Mechanics, 8082 Penn State University, University Park, Pennsylvania 16802, United States; ‡ Department of Inorganic Chemistry, University of Chemistry and Technology Prague, Prague 16000, Czech Republic; § 2DCC, Penn State University, University Park, Pennsylvania 16802, United States; ∥ Materials Science and Engineering, Penn State University, University Park, Pennsylvania 16802, United States; ⊥ Department of Physics, 92991 Harcourt Butler Technical University, Kanpur, Uttar Pradesh 208002, India; # Electrical Engineering, Penn State University, University Park, Pennsylvania 16802, United States

**Keywords:** van der Waals thiophosphate, 2D materials, opto-ionic-electronic coupling, fading memory

## Abstract

In conventional field-effect transistors (FETs), the gate dielectric primarily provides electrostatic control of the channel and is engineered to remain chemically and electronically inert to ensure stable operation. Emerging studies, however, suggest that functional dielectrics integrated with two-dimensional (2D) semiconductors can introduce additional physical mechanisms that influence device behavior. Here, we investigate AgInP_2_S_6_ (AIPS), a high-κ van der Waals thiophosphate, as a functional top-gate dielectric in MoS_2_ field-effect transistors. The AIPS/MoS_2_ heterointerface exhibits coupled opto-ionic-electronic interactions, where defect-mediated sub-bandgap photoactivity and photon-assisted Ag^+^ ion migration independently occur within the dielectric yet collectively modulate the channel conductance. Optical excitation redistributes charge within the dielectric, gradually reconfiguring the ionic landscape and altering the interfacial electrostatic potential. The resulting conductance exhibits strong temperature dependence. At low temperatures (∼0 °C), reduced phonon activity suppresses Ag^+^ mobility, leading to persistent photoconductivity. At elevated temperatures (∼80 °C), thermally activated ion hopping accelerates relaxation of the stored state. This temperature-dependent retention behavior enables a thermally tunable fading memory response, where the persistence of the optically induced conductance state can be controlled through ionic dynamics. These results highlight how opto-ionic coupling at 2D/dielectric interfaces can introduce stimulus-responsive functionality in transistor architectures, providing a platform for devices that combine sensing and memory behavior within the gate stack.

## Graphical abstract

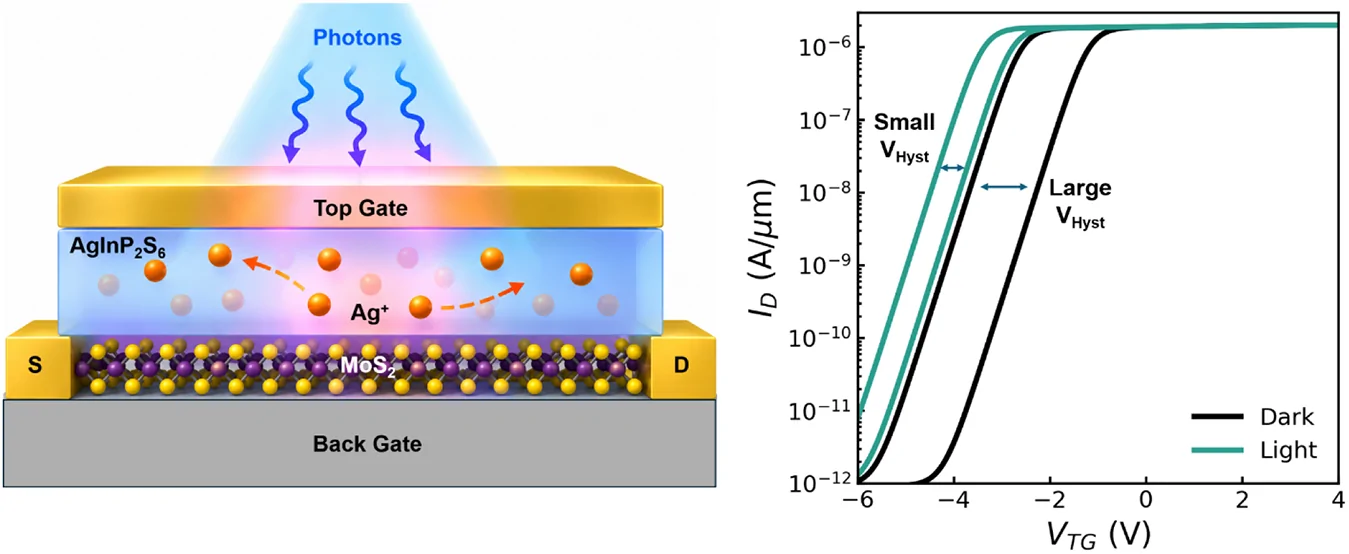

## Introduction

The growing demand for compact, intelligent sensors for environmental monitoring,^1–3^ wearable electronics,^4–6^ and the Internet of Things (IoT)^7^ is driving the development of miniaturized devices capable of sensing, processing, and adapting to multiple physical stimuli such as temperature, light, and chemical cues. Conventional approaches to achieving such functionality often rely on the heterogeneous integration^8,9^ of separate sensing, storage, and processing components, which increases system complexity, power consumption, and footprint. Here, we explore an alternative approach in which the gate dielectric in a field-effect transistor (FET) is not strictly passive. By leveraging the unique properties that emerge at the interfaces of van der Waals (vdW) heterostructures formed from two-dimensional (2D) materials,^10–13^ the dielectric can participate more actively in device behavior, influencing sensing, memory, and computing functionalities.^14,15^

In conventional field-effect transistors (FETs), the gate dielectric is designed to be an electrostatically active, yet physically inert component. Its primary role is to modulate the channel charge density while remaining unresponsive to environmental stimuli, such as temperature or light. Additionally, dielectrics are highly desirable to be devoid of mobile ions to ensure operational stability and reliability.^16–21^ This design philosophy, central to the success of silicon complementary metal oxide semiconductor (CMOS) technology, has enabled decades of scaling and performance gains by preserving the dielectric as a passive, predictable element. However, this same passivity also limits functionality: the dielectric contributes nothing beyond static electrostatic control, forcing sensing, memory, and signal processing functions to be realized through additional materials, layers, and circuits. Such segregation of roles not only increases complexity, power consumption, and footprint but also constrains the realization of truly multifunctional device architectures.

Furthermore, recent studies on CuInP_2_S_6_ (CIPS) demonstrate that layered metal thiophosphates naturally exhibit opto-ionic coupling arising from the interplay between mobile cations and photogenerated carriers. In CIPS photodetectors, illumination generates electron–hole pairs while simultaneously activating Cu^+^ ion hopping within the weakly bonded van der Waals lattice, leading to a tunable photoresponse that depends on illumination intensity and wavelength. Under moderate illumination, photon-assisted ionic migration enhances conductivity, whereas stronger illumination can neutralize mobile Cu^+^ ions through recombination with photogenerated electrons, thereby modifying the ionic transport and device conductance.^22^ Complementary studies on ferroionic CIPS heterostructures further confirm that field-driven Cu migration can modulate the electronic transport characteristics of the device, establishing the coupled ionic-electronic nature of this material family.^23^ Given the close structural and chemical similarity between CuInP_2_S_6_ and AgInP_2_S_6_ where Ag^+^ ions occupy analogous weakly bound sites within the (P_2_S_6_) framework, a similar ionic mobility is expected in AgInP_2_S_6_.

In this context, we explore the possibility that the gate dielectric can play a more active role in transistor operation when its intrinsic material properties are carefully utilized. In particular, the dielectric can contribute to sensing, memory, and computing functionalities through additional physical processes beyond purely electrostatic gating. We investigate this concept using AgInP_2_S_6_ (AIPS), a high-κ van der Waals (vdW) thiophosphate, integrated as the top-gate dielectric in MoS_2_ field-effect transistors. AIPS uniquely combines defect-mediated sub-bandgap photoactivity^24^ with temperature-assisted Ag^+^ ion migration, two processes that are intrinsically independent yet converge at the AIPS/MoS_2_ heterointerface to modulate channel transport. Optical excitation redistributes charge within the dielectric, initiating ionic reconfiguration, while the temperature governs the persistence of this state. At low temperatures, suppressed phonon activity limits Ag^+^ mobility, producing persistent photoconductivity, whereas at higher temperatures enhanced phonon-assisted migration accelerates relaxation of the photoinduced state and restores the initial conductance. This temperature-dependent retention and relaxation behavior gives rise to a thermally tunable fading memory, where the persistence of the stored state is governed by ionic dynamics within the dielectric. More broadly, the coupling of photons, ions, and electrons within a vdW dielectric highlights how functional gate dielectrics can introduce stimulus-responsive behavior at the device level, expanding the role of the gate stack beyond purely electrostatic control toward sensing and memory functionalities.

## Results

### Structural and Dielectric Properties of AgInP_2_S_6_ (AIPS)

AIPS, synthesized via chemical vapor transport (CVT), is a layered vdW thiophosphate that crystallizes in a trigonal lattice (space group: *P*3̅1*c*).^24,25^ As shown in Figure 1a, its crystal structure comprises stacked layers of Ag, In, and [P_2_S_6_] units held together by vdW forces, enabling facile mechanical exfoliation. An optical micrograph of a representative AIPS crystal is shown in Figure 1b, illustrating the silver-gray, lamellar AIPS crystals with lateral dimensions reaching several millimeters. Supplementary Information 1 shows an atomic force microscopy (AFM) image of a representative AIPS flake revealing a uniform thickness of 36 nm with minimal surface roughness over an extended area. The out-of-plane structural ordering was examined by X-ray diffraction (XRD), as shown in Figure 1c. The presence of sharp (00l) reflections confirms the high crystallinity and preferred layer stacking along the c-axis. Furthermore, the chemical composition and bonding states were examined by X-ray photoelectron spectroscopy (XPS) as depicted in Figure 1d-h, which revealed characteristic peaks for Ag 3d, In 3d, P 2p, and S 2p, consistent with a sulfide-rich [P_2_S_6_]^4–^ framework and confirming phase purity. Notably, the Ag 3d_5/2_ peak at 368.2 eV verifies the presence of monovalent Ag­(I) or Ag^+^ ions, in agreement with previous reports.^25^ Additionally, the optical characteristics of AIPS have been displayed in Figure 1i. Raman spectroscopy was performed on a representative AIPS flake, and the Raman spectrum exhibits four well-resolved vibrational modes associated with the internal dynamics of the [P_2_S_6_] unit, including S–P–P and S–P–S deformations, P–P symmetric stretching, and P–S oscillations. Subtle variations in the P–P stretching mode suggest minor lattice distortions, potentially linked to thermally activated cation (Ag^+^) migration.

**Figure 1:**
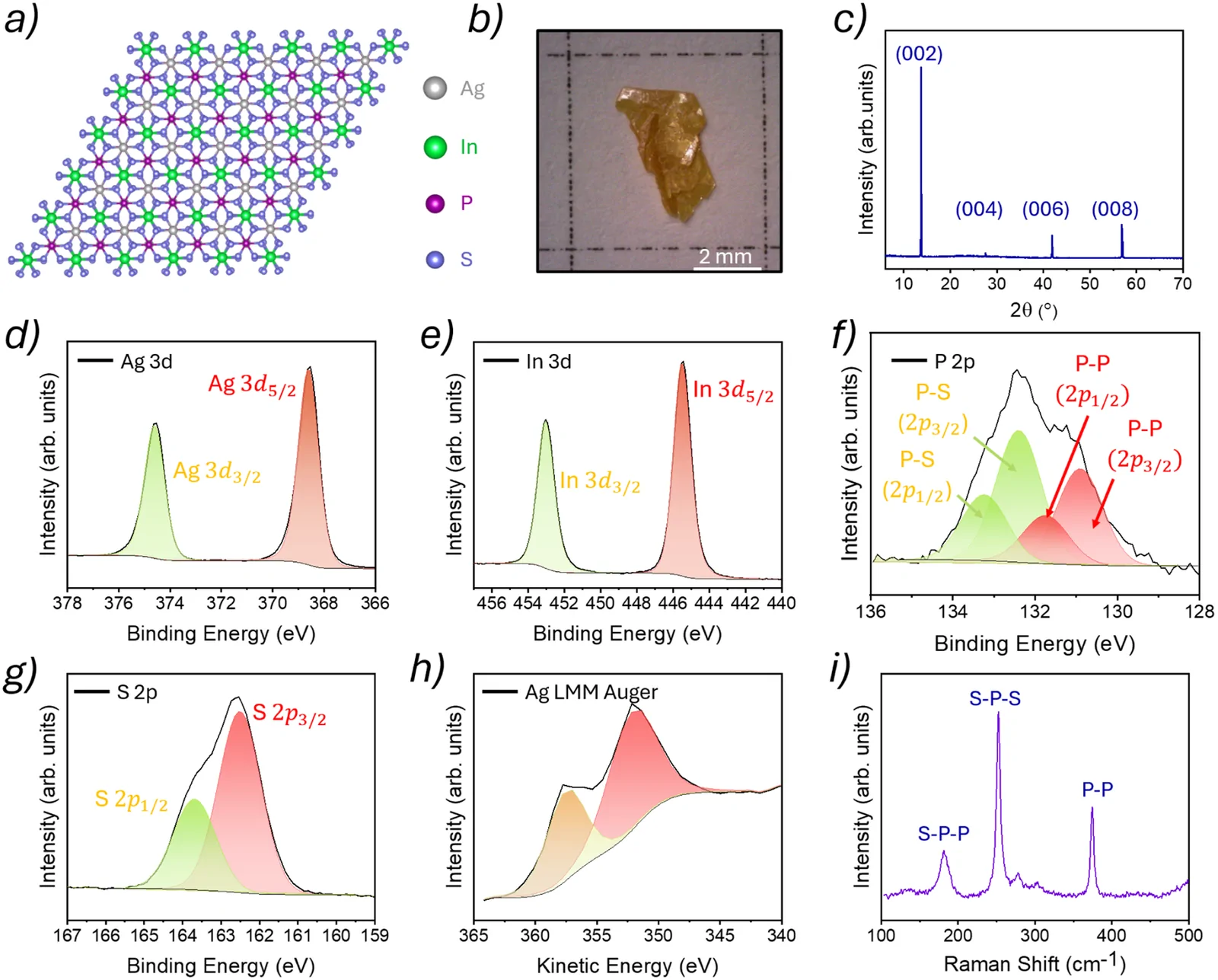
Structural and compositional characterization of AgInP_2_S_6_ (AIPS). (a) Crystal structure of AgInP_2_S_6_ (AIPS) viewed along the *c*-axis. (b) Optical micrograph of a bulk AIPS crystal. (c) X-ray diffraction (XRD) pattern displaying prominent (00l) reflections, confirming phase purity and preferred layered orientation. (d-h) X-ray photoelectron spectroscopy (XPS) core-level spectra of Ag 3d, In 3d, P 2p, S 2p, and the Ag LMM Auger peak, confirming the expected chemical composition; the Ag 3d_5/2_ peak at 368.2 eV indicates the presence of monovalent Ag^+^. (i) Raman spectrum acquired with a 532 nm excitation laser, showing four characteristic vibrational modes of the [P_2_S_6_] unit corresponding to S–P–P and S–P–S deformations, P–P symmetric stretching, and P–S vibrations. Minor shifts in the P–P stretching mode suggest local lattice distortions that may be associated with Ag-related defects or cation dynamics.

Photoluminescence (PL) measurements displayed in Supplementary Information 2a reveal a prominent emission peak at ∼1.9 eV, which is notably lower than the optical bandgap of ∼2.39 eV.^24^ This clearly indicates that the PL emission originates from defect states within the bandgap rather than from band-to-band transitions.^26^ The sub-bandgap PL emission can be attributed to the presence of point defects such as sulfur vacancies,^27^ which introduce localized trap states that can capture photogenerated carriers. These trapped carriers undergo radiative recombination with valence band electrons, resulting in the emission of photons with sub-bandgap energies. Moreover, the schematic in Supplementary Information 2b illustrates the excitation and relaxation pathways of photocarriers in AIPS, highlighting the role of defect-mediated optical absorption and emission processes.

Next, to evaluate the dielectric strength of AIPS, we fabricated dual-gated FETs using monolayer MoS_2_ as the semiconducting channel, as schematically illustrated in Figure 2a. The back gate consisted of a 25 nm Al_2_O_3_ dielectric grown by atomic layer deposition (ALD) on a Ti/Pt (10/30 nm) electrode stack. Exfoliated AIPS flakes were then integrated as the top-gate dielectric, while Ni/Au served as the top-gate metal as well as the source–drain contacts. A scanning electron microscope (SEM) image of a representative dual-gated MoS_2_ FET (channel length *L*_CH_ = 1 μm and channel width, *W*_CH_ = 1 μm) is shown in Figure 2b. Detailed fabrication procedures are described in the Methods section. Supplementary Information 3 depicts the back-gate transfer characteristics, i.e., drain-to-source current (*I*_DS_) as a function of the back-gate voltage (*V*_BG_) at a constant drain-to-source bias, *V*_DS_ = 1 V for multiple MoS_2_ FETs. As expected, all devices exhibit n-type conduction with a large current on/off ratio. Figure 2c shows top-gate transfer characteristics for a representative AIPS-gated MoS_2_ FET obtained by sweeping *V*_TG_ at a constant *V*_BG_ = 4 V and *V*_DS_ = 1 V, exhibiting an on–off current ratio (*I*_ON_/*I*_OFF_) of >10^7^ alongside a steep subthreshold slope (SS). Supplementary Information 4 shows the top-gate transfer characteristics for a representative AIPS-gated MoS_2_ FET obtained by sweeping the *V*_TG_ and stepping the *V*_BG_ from 5 to −2 V. Under these conditions, the back gate modulates the entire channel, including regions beneath the contacts where carrier injection occurs, thereby determining the on-state current.

**Figure 2:**
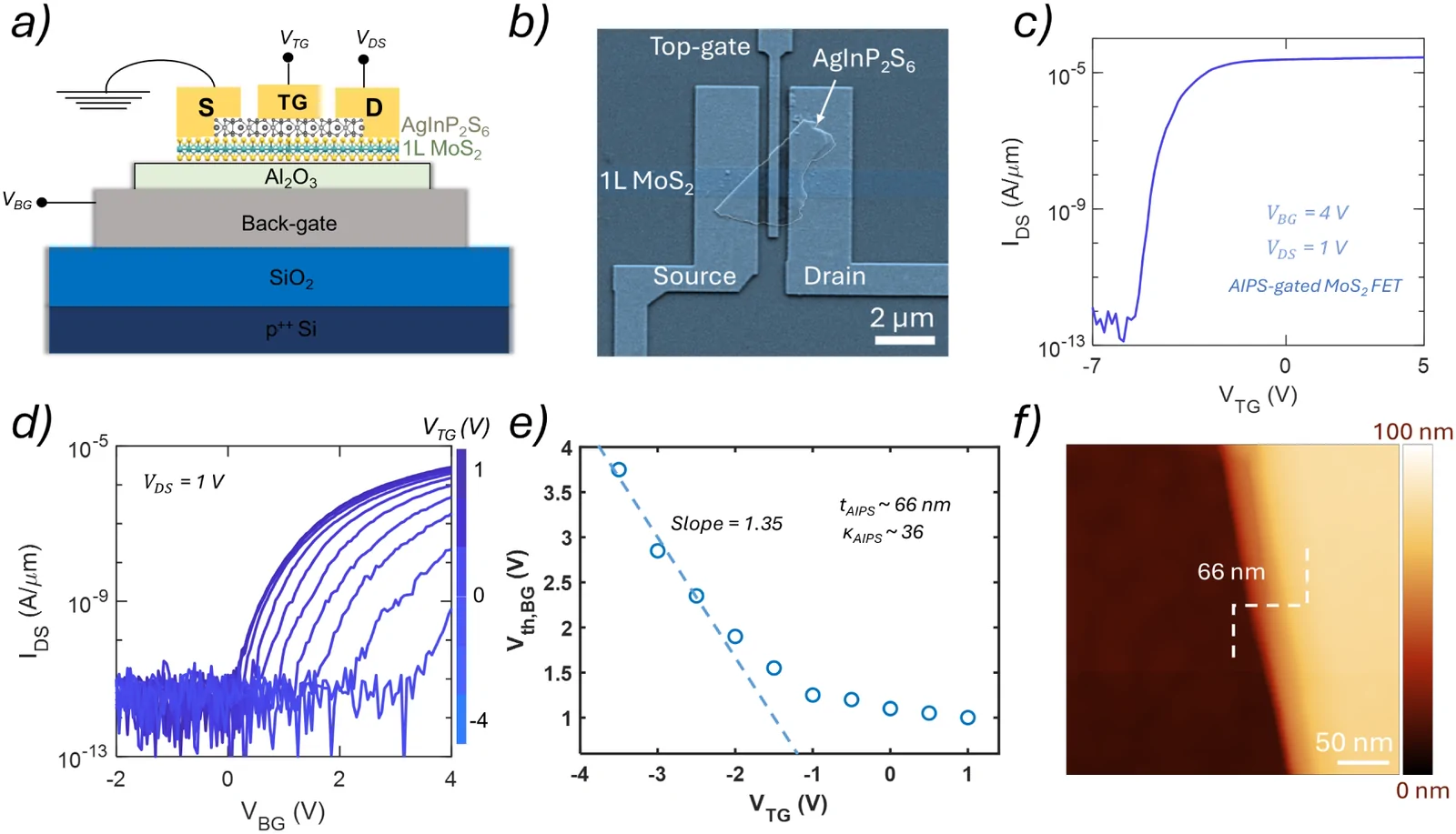
AIPS as a top-gate dielectric in monolayer MoS_2_ FETs. (a) Schematic illustration and (b) scanning electron microscopy (SEM) image of a dual-gated MoS_2_ FET employing exfoliated AIPS as the top-gate dielectric. (c) Top-gate transfer characteristics of the AIPS-gated MoS_2_ FET obtained by sweeping the top-gate voltage *V*_TG_ from −7 V to 5 V at fixed *V*_BG_ = 4 V and *V*_DS_ = 1 V. (d) Back-gate transfer characteristics (*I*_DS_ versus *V*_BG_) measured at *V*_DS_ = 1 V for different *V*_TG_ values. (e) Back-gate threshold voltage *V*_th,BG_, extracted using the iso-current criterion *I*_DS_ = 10 nA μm^–1^, plotted as a function of *V*_TG_. The linear dependence reflects the capacitive coupling between the gates, where the slope corresponds to the ratio of the back-gate and top-gate equivalent oxide thicknesses (EOTs), yielding an effective dielectric constant of ∼36 for a ∼66 nm thick AIPS flake. (f) Atomic force microscopy (AFM) image of an exfoliated AIPS flake used as the top-gate dielectric, showing a thickness of approximately 66 nm.

In contrast, the partially overlapping top gate locally depletes carriers in the channel region, enabling the device to turn off under appropriate bias conditions. Notably, even at the highest *V*_BG_ = 5 V, the top gate fully depletes the channel, achieving *I*_ON_/*I*_OFF_ > 10^7^, confirming that AIPS can electrostatically modulate *I*_DS_ and act as an active dielectric gate. Additionally, the top-gate leakage current (*I*_TG_) remains below 10^–3^ A/cm^2^ across the entire *V*_TG_ range as shown in Supplementary Information 5, demonstrating the excellent insulating quality and breakdown robustness of exfoliated AIPS layers.

To quantify the dielectric properties of AIPS, we next extracted its effective gate capacitance by measuring the dual-gated transfer characteristics. Back-gate transfer characteristics were measured while stepping the top-gate voltage (*V*_TG_), as shown in Figure 2d. The corresponding back-gate threshold voltages (*V*_th,BG_) were extracted using an iso-current criterion of *I*_DS_ = 10nA μm^–1^. Plotting *V*_th,BG_ as a function of *V*_TG_ (Figure 2e) yields a linear dependence in the depletion regime, where the slope corresponds to the capacitance ratio *C*_BG_/*C*_TG_.^28,29^ Using the known *C*_BG_ ≈ 3.3 × 10^–3^ Fm^–2^ for 25 nm thick Al_2_O_3_, we estimate *C*_TG_ ≈ 2.45 × 10^–3^ Fm^–2^ for the AIPS gate stack. AFM measurements indicate an AIPS thickness of ∼66 nm for the corresponding device as shown in Figure 2f, from which we deduce a relative dielectric constant κ ≈ 36, surpassing many conventional high-κ dielectrics. Supplementary Information 6 shows the relative dielectric constant across multiple devices, yielding comparable κ values of ≈39 and ≈30 for AIPS flakes with thicknesses of 58 and 74 nm, respectively. These results establish AIPS as a high-quality, high-κ vdW gate dielectric^30–33^ capable of delivering strong electrostatic control with low leakage in dual-gated 2D FET architecture.

### Evidence of Temperature-Assisted Ag^+^ Ion Migration in AIPS-Gated MoS_2_ FET

In conventional FETs, the presence of mobile ions in the gate dielectric is highly undesirable, as it leads to threshold voltage drift, hysteresis, and reliability concerns. Dielectrics are therefore engineered to be chemically stable and free of ionic species. In contrast, multiple ionic species may be present in AIPS; however, Ag^+^ ions are considered the most probable mobile species within the lattice, providing a platform where ion migration may be harnessed as a functional mechanism. The presence of Ag^+^ in AIPS is confirmed by X-ray photoelectron spectroscopy (XPS), which shows a distinct Ag 3d^5/2^ peak at 368.2 eV, consistent with monovalent Ag­(I). Given the layered crystal structure of AIPS, where Ag^+^ ions reside between covalently bonded $[P_{2}S_{6}{]}^{4-}$ units, the cations experience relatively weak electrostatic confinement compared to the rigid thiophosphate framework. The layers are separated by van der Waals gaps, and the Ag sites form partially open coordination environments within the lattice. As a result, the energy barrier for cation displacement is expected to be relatively low, making Ag^+^ ions the most likely candidates for field-driven migration. Consequently, under an applied electric field or external stimuli, these Ag^+^ ions can redistribute within the lattice, providing a plausible pathway for ionic motion that can influence the electrostatic environment at the AIPS/MoS_2_ interface. This property is fundamentally different from conventional oxide dielectrics and opens the possibility of coupling ionic motion to electronic transport in a transistor channel. Dual-sweep top-gate transfer characteristics of AIPS-gated MoS_2_ FETs shown in Figure 3a reveals a pronounced counterclockwise (CCW) hysteresis, which is direct experimental evidence of mobile ionic species influencing the channel electrostatics.^34^ Unlike ferroelectric switching,^35,36^ here the forward and reverse sweeps display asymmetric subthreshold slopes: the forward sweep is steeper, while the reverse sweep is more gradual. This asymmetry arises from the time-lagged redistribution of Ag^+^ ions relative to the applied gate field, producing a dynamic internal potential that modulates the channel conductance. Supplementary Information 7 shows dual-sweep top-gate transfer characteristics for several AIPS-gated MoS_2_ FETs displaying CCW hysteresis, indicating the presence of Ag^+^ ions in the dielectric layer.

**Figure 3:**
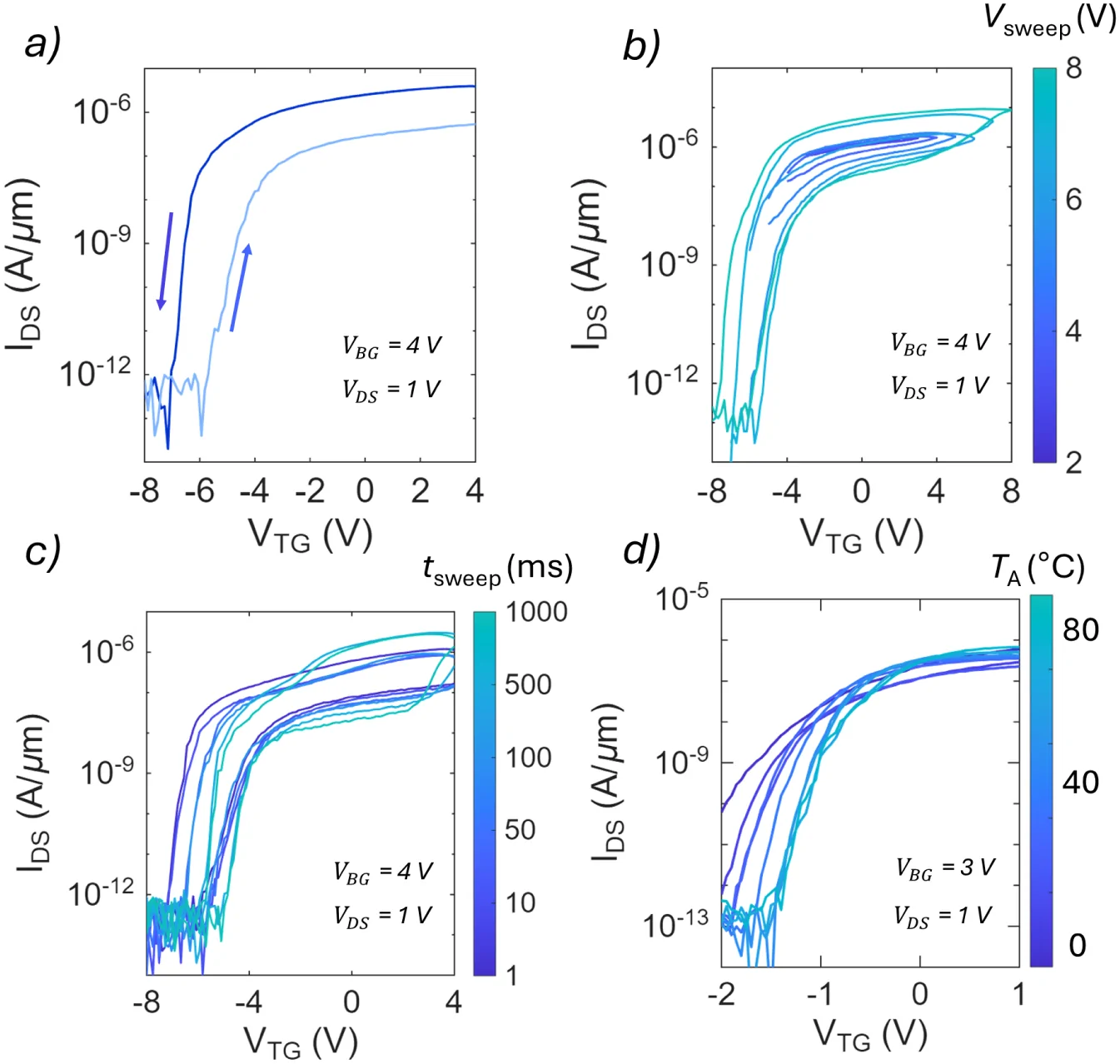
Ion migration dynamics in AIPS-gated MoS_2_field-effect transistors. (a) Counterclockwise hysteresis at dual-sweep top-gated transfer characteristics of AIPS-gated MoS_2_ FETs. (b) Top-gated transfer characteristics at different sweep ranges of *V*_TG_ showcasing the minor hysteresis loops in smaller *V*_TG_ range. (c) Dual-sweep top-gate transfer characteristics, i.e., *I*_DS_ measured by varying sweep rates (*t*_sweep_ : 1 ms to 1 s) while keeping the *V*_BG_ fixed for AIPS-gated MoS_2_ FETs. (d) Temperature-dependent top-gate transfer characteristics, measured across different temperatures, *T*_A_ for AIPS-gated MoS_2_ FETs. Note that the transfer characteristics show that hysteresis increases with increasing sweep duration and the on/off ratio increases with increasing temperature (*T*_A_). Increasing temperature and sweep time allow enhancement of ion mobility and its redistribution during each sweep cycle. This leads to improvements in on/off ratios for AIPS-gated MoS_2_ FETs.

Further confirmation comes from sweep-range-dependent measurements presented in Figure 3b. When *V*_TG_ is cycled over a reduced voltage range, the transfer curves exhibit minor hysteresis loops, reflecting incomplete Ag^+^ redistribution. This history-dependent electrostatic modulation is absent in inert dielectric-gated FETs. Additionally, sweep-time-dependent measurements shown in Figure 3c provide kinetic insight: slower voltage sweeps produce larger hysteresis windows (*V*_Hyst_), consistent with more complete ionic redistribution over longer times. Here, *V*_Hyst_ is defined as the difference between the threshold voltages extracted from the forward and reverse top-gate sweeps, determined using an iso-current criterion of *I*_DS_ = 10 nA μm^–1^. Faster sweeps reduce *V*_Hyst_, indicating that the electrostatic modulation is limited by Ag^+^ mobility compared to the field-sweep time. Finally, temperature-dependent measurements presented in Figure 3d show a clear increase in the on/off current ratio between 0 °C and 80 °C under identical sweep conditions. At low temperatures, reduced phonon population suppresses Ag^+^ hopping, limiting ionic modulation of the channel. At elevated temperatures, enhanced phonon activity lowers the activation barrier for migration, enabling more complete redistribution within the gate sweep. This establishes a temperature-assisted Ag^+^ migration mechanism directly linking lattice vibrations to ionic-electronic coupling in the AIPS/MoS_2_ heterostructure. Such strong, temperature-tunable ionic-electronic interactions are unprecedented in conventional dielectric-gated FETs, where purely electronic effects dominate channel control.

### Defect-Mediated Photoactivity and Opto-Ionic Coupling in AIPS-Gated MoS_2_ FET

Having established AIPS both as a high-κ top-gate dielectric and as a medium supporting temperature-regulated Ag^+^ migration, we next investigate its defect-mediated optical response and the resulting opto-ionic coupling in an AIPS/MoS_2_ vdW heterostructure. Supplementary Information 8 shows the dual-sweep top-gate transfer characteristics of a representative device under dark conditions and under illumination (*P*_in_ = 5 mW/cm^2^). We observe three distinct trends under illumination: an enhancement in the on-state current (*I*_ON_), a negative shift in *V*_th,TG_, and a decrease in the *V*_Hyst_. The first two observations are consistent with photogenerated holes being trapped within the AIPS dielectric and electrons transferred to the MoS_2_ channel, thereby reinforcing its n-type conduction. The origin of this response lies in the band alignment between AIPS and MoS_2_. As shown in Supplementary Information 9, sub-bandgap defect states in AIPS are located near its conduction band edge, closely aligned with the MoS_2_ conduction band minima. Upon illumination, this band alignment promotes the trapping of photogenerated holes within AIPS, while facilitating efficient electron transfer into the MoS_2_ conduction band. Furthermore, we carried out density functional theory (DFT) calculations to quantify the defect-mediated photoresponse in AIPS-gated 2D FETs. These simulations provide a deeper understanding of the defect states, offering a direct microscopic correlation with the experimentally observed photoresponse characteristics of AIPS-gated MoS_2_ FETs. Furthermore, the defect landscape in AIPS is likely governed by Ag vacancies and/or S vacancies as presented in Supplementary Information 10, which exhibit comparatively lower formation energies of ∼0.1 eV and ∼0.26 eV, respectively. Nevertheless, these defects shift the Fermi level (*E*_f_) closer to the conduction band edge of AIPS, creating localized donor-like states. Upon illumination, these states facilitate the generation of electron–hole pairs; the photogenerated holes remain trapped within AIPS, while the electrons are injected into the conduction band of MoS_2_. This charge transfer increases the channel carrier density, leading to a rise in drain current and a negative shift in threshold voltage. As a result, AIPS-gated MoS_2_ FETs exhibit a pronounced positive photoresponse, consistent with enhanced photoconductivity under illumination. Control experiments using back-gated MoS_2_ devices without an AIPS top dielectric (Supplementary Information 11) show negligible photoresponse under identical illumination, consistent with the weak optical absorption of monolayer MoS_2_ ^37,38^ at low light intensities. This confirms that the observed photoconductivity originates from defect-assisted charge transfer in the AIPS dielectric rather than intrinsic MoS_2_ photophysics. Finally, the third observation indicates that photoexcitation not only alters the interfacial electrostatics via trapped charges but also accelerates Ag^+^ migration, likely through localized photothermal heating or light-induced modulation of the internal field, leading to greater ionic displacement during gate sweeps.

Temperature-dependent measurements further substantiate the presence of opto-ionic coupling in AIPS-gated devices. Figure 4a and b, respectively, show the evolution of dual-sweep top-gate transfer characteristics of an AIPS-gated MoS_2_ FET in dark and under illumination (*P*_in_ = 5 mW/cm^2^) as the ambient temperature (*T*_A_) is increased from 0 °C to 80 °C, with a step of 20 °C. The extracted *V*_Hyst_ values for both conditions are plotted against *T*_A_ in Figure 4c-d. We also demonstrate the dual-sweep top-gate transfer characteristics of an AIPS-gated MoS_2_ FET in dark and under illumination measured for the intermediate *T*_A_ values in Supplementary Information 12. In the absence of illumination, the device’s behavior is dominated by the slow migration of cations within the AIPS lattice. Under an applied gate bias, these mobile ions undergo field-driven drift toward the AIPS/MoS_2_ interface, effectively functioning as a “sluggish” internal gate. Because the ionic relaxation time is orders of magnitude longer than the intrinsic electronic response time of the MoS_2_ channel, the system develops a pronounced electrostatic lag. This manifests as remanent interfacial charge redistribution, leading to a substantial threshold voltage shift (*V*_th_) and large *V*_Hyst_ during dual-sweep transfer measurements. In the dark, increasing temperature enhances Ag^+^ mobility, allowing ions to drift and relax more efficiently during the gate sweep. When the ionic relaxation time becomes comparable to or shorter than the sweep time scale, the electrostatic lag between forward and reverse sweeps is reduced, leading to a narrowing of the hysteresis window. Notably, *V*_Hyst_ exhibits a non-monotonic temperature dependence, rising steadily up to *T*_A_ = 50 °C before decreasing sharply at higher temperatures. This trend reflects the competition between ionic mobility and ionic retention. At even higher *T*_A_, ionic motion becomes so fast that the system remains near equilibrium throughout the sweep, reducing the lag and diminishing *V*_Hyst_. This non-monotonic temperature dependence reflects the balance between thermally activated ion transport and the time constants for ionic relaxation relative to the measurement time scale.

**Figure 4:**
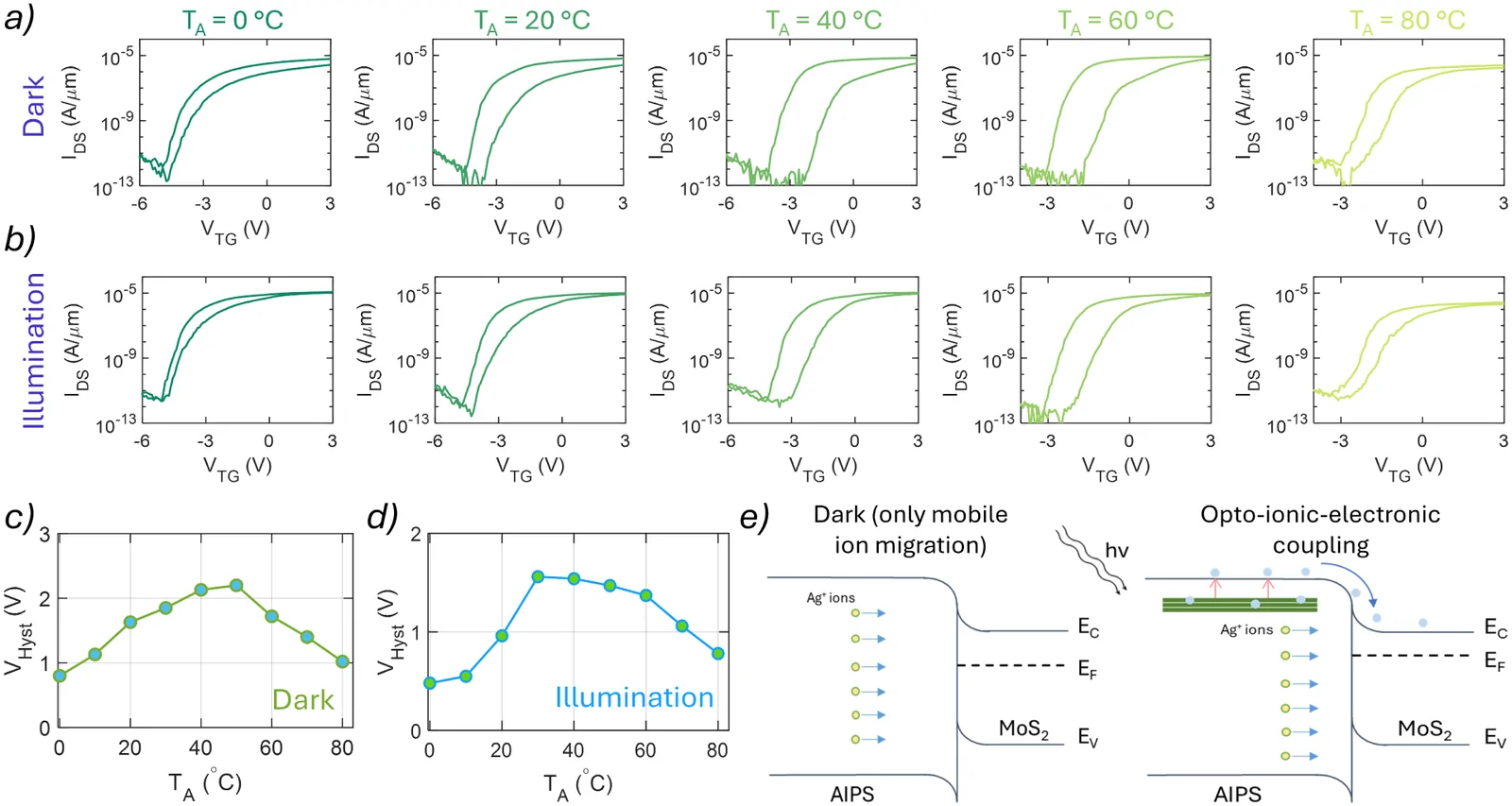
Opto-ionic coupling in AIPS-gated MoS_2_ FETs. Top-gate transfer characteristics of MoS_2_ FETs measured at different temperatures (*T*_A_ = 0–80 °C) under dark (a) and (b) illuminated conditions. Extracted hysteresis window (*V*_Hyst_) as a function of *T*_A_ in (c) dark and (d) under illumination. (e) Band diagrams illustrating the mechanisms: in the dark, Ag^+^ ion migration in AIPS shifts the channel potential, while under illumination, photoexcited carriers introduce additional opto-ionic-electronic coupling that modulates the MoS_2_ band edges.

Upon illumination with photons of energy exceeding the AIPS bandgap (*hv* > *E*_g_), electron–hole pairs are generated within the dielectric, and a dynamic charge redistribution occurs. Photogenerated holes are preferentially trapped within the AIPS bulk or at defect sites, where they partially screen the electrostatic field associated with accumulated Ag^+^ ions. Simultaneously, photogenerated electrons are transferred across the AIPS/MoS_2_ heterointerface into the MoS_2_ channel, increasing the carrier density via a photogating mechanism and enhancing the on-state current. This rapid electronic compensation effectively counteracts the slower ionic displacement, reducing the internal field asymmetry responsible for hysteresis. As a result, the *V*_Hyst_ collapses significantly under illumination. As *T*_A_ rises, the combined effect of enhanced ionic mobility and photoassisted charge redistribution drives the system closer to quasi-equilibrium during the sweep, resulting in a more pronounced collapse of *V*_Hyst_. Figure 4e presents the band alignment between AIPS and MoS_2_ under dark and illumination, illustrating the optical and ionic coupling. This opto-ionic interaction dynamically shifts the interfacial potential, demonstrating the synergistic modulation of the band structure by both photoexcitation and ionic migration.

Furthermore, we have expanded the experimental evidence to substantiate the ion migration mechanism under dark and illuminated conditions. Sweep-time-dependent transfer characteristics were measured at different temperatures to quantify the thermally activated migration process^39^ for Ag^+^ ions. Supplementary Informations 13–14 present the dual-sweep top-gate transfer characteristics of MoS_2_ FETs gated with AIPS, respectively, measured over sweep durations (*t*_sweep_) ranging from 10 μs to 1 s and at different temperatures (*T* = 25, 50, 75, and 100°C). The corresponding hysteresis window, *V*_Hyst_, defined as the voltage difference between the forward and reverse sweeps at a fixed current level, is plotted as a function of *t*_sweep_ in Supplementary Informations 13–14. Notably, for AIPS-gated MoS_2_ FETs, the *V*_Hyst_ exhibits a non-monotonic dependence on *t*_sweep_, displaying a pronounced maximum at a characteristic time *t*_peak_. With increasing temperature, *t*_peak_ systematically shifts toward shorter times, consistent with thermally activated ion migration, while the maximum value of *V*_Hyst_ remains nearly unchanged.

To gain quantitative insight into the migration energetics, we constructed an Arrhenius plot using the temperature-dependent shift in *t*_peak_ (Supplementary Informations 13–14). Here, *t*_peak_ represents the characteristic time required for Ag^+^ ions to drift across the full dielectric thickness (*d*_AIPS_) under the applied electric field, as described by eq 1:

1 $$\frac{1}{t_{\mathrm{peak}}}=\frac{1}{\left(\frac{D_{c}E_{i}}{d_{\mathrm{AIPS}}}\right)\frac{\left|q\right|}{k_{B}T}\exp\left(-\frac{E_{a}}{k_{B}T}\right)}$$

where *E_i_* denotes the time-averaged electric field, *E*_a_ is the effective activation energy for ion migration, *D*_c_ is the effective diffusion coefficient, *q* is the electronic charge, and *k*_B_ is Boltzmann’s constant. From the slope of the Arrhenius plot, the effective activation energy for ionic migration was extracted to be ∼0.53 eV under dark conditions (*P*_in_ = 0 mW cm^–2^). Under illumination (*P*_in_ = 5 mW cm^–2^), the extracted effective activation energy decreases to ∼0.27 eV, indicating that optical excitation facilitates ionic motion in AIPS-gated MoS_2_ FETs. This reduction in *E*_a_ suggests that photoexcitation modifies the local electrostatic landscape, likely through photogenerated charge redistribution within the dielectric, thereby lowering the effective migration barrier.

Although multiple ionic species may be present in AIPSs, Ag^+^ ions are considered the most probable mobile species due to their relatively weak electrostatic confinement within the layered thiophosphate framework and their partially open coordination environment. In contrast, In and the (P_2_S_6_) backbone form a more rigid covalent network, making their displacement energetically less favorable. Therefore, the observed reduction in the effective activation energy under illumination is most consistently attributed to photon-assisted Ag^+^ migration, where light-induced electronic excitation lowers the effective hopping barrier for Ag^+^ ions. These findings reinforce the ionic origin of hysteresis behavior and establish a quantitative link among light-induced defect modulation, ion transport, and hysteresis dynamics in AIPS-based 2D FETs.

To elucidate further on the ion migration dynamics, we also performed time-resolved *V*_th_ evolution experiments at constant bias and temperature to determine ion mobilities and diffusion coefficients, under dark and illuminated conditions for different temperatures, using a drift-diffusion model. For reference, the initial back-gate transfer characteristics of the 2D FETs, along with a theoretical fit based on the virtual source (VS) model,^38,40^ are shown in Supplementary Information 15. Notably, the *I*_DS_ of a representative AIPS-gated MoS_2_ FETs measured over time at a constant *V*_BG_ of 3 V and *V*_DS_ of 1 V with a *V*_TG_ of 3 V as shown in Supplementary Informations 16–17. Over time, Ag^+^ ions migrate within the AIPS dielectric, leading to a gradual shift in the threshold voltage $\left(V_{th_{p}}\right)$ , manifested as a monotonic increase in the drain current (*I*_DS_). The threshold voltage extracted from this fit was subsequently monitored as a function of time using the data, and its temporal evolution is presented in Supplementary Informations 16–17.

2 $$V_{th_{p}}=V_{th_{i}}-\frac{Q_{ion}}{C_{AIPS}}\frac{X_{ion}}{t_{AIPS}}$$

In eq 2, $V_{th_{i}}$ represent the threshold voltage of the back-gate without any mobile ions in the top-gate dielectric, *Q*_ion_ is the mobile ion charge, *X*_ion_ is the centroid of the mobile ion charge distribution, *C*_AIPS_ is the capacitance of the top-gate dielectric, and *t*_AIPS_ is the thickness of the top-gate dielectric. Finally, the centroid of the mobile ion charge distribution (*X*_ion_) was determined according to eq 3. Supplementary Informations 16–17 show *X*_ion_ as a function of time alongside a fit created using eq 3 listed below.

3 $$X_{ion}=\sqrt{{\left(\mu_{ion}Et\right)}^{2}+D_{ion}t}$$

Here, μ_ion_ is the apparent mobility of Ag^+^ ions, E=VAIPStAIPS is the electric field, and *D*_ion_ is the effective diffusion coefficient of the mobile ions. We found that μ_ion_ = 4.3 × 10^–16^ m^2^ V^–1^ s^–1^ and 7.1 × 10^–16^ m^2^ V^–1^ s^–1^ for Ag^+^ ions, under dark for *T* = 25 and 50 °C. Also, the *D*_ion_ was found to be 9 × 10^–19^ m^2^ s^–1^ for Ag^+^ ions. Notably, the Ag^+^ ion mobility increases to μ_ion_ = 5 × 10^–16^ m^2^ V^–1^ s^–1^ and 8.5 × 10^–16^ m^2^ V^–1^ s^–1^ under light for *T* = 25 and 50 °C, as depicted in Supplementary Information 18. Similar results were obtained for *D*_ion_ under dark and illuminated conditions for *T* = 25 and 50 °C as displayed in Supplementary Information 18. Upon illumination, photoexcited carriers interact with the local defect landscape, enhancing the drift of Ag^+^ ions within the lattice. This photoassisted ionic motion accelerates the redistribution of Ag^+^ ions at the AIPS/MoS_2_ interface, leading to a faster and more pronounced modulation of the channel conductance. It should be noted that variations in ionic mobility do not necessarily correspond directly to proportional changes in the extracted effective activation energy. The reported *E*_a_ values (Supplementary Informations 13–14) represent an effective parameter describing the dominant migration process within the dielectric under a given condition. Under illumination, photogenerated carriers can modify the local electrostatic environment and defect landscape, facilitate ionic motion, and thereby reducing the effective migration barrier. Nevertheless, the observed enhancement in Ag^+^ mobility under light thus provides direct evidence of opto-ionic coupling, where photonic excitation and ionic dynamics are intrinsically linked to govern the electrical response of AIPS-gated 2D FETs.

Additionally, we evaluated the operational stability of the AIPS-gated MoS_2_ FETs by repeatedly measuring the top-gate transfer characteristics over multiple consecutive cycles and across different days, as shown in Supplementary Information 19a. The transfer curves exhibit minimal variation in threshold voltage, subthreshold behavior, and on-state current, indicating excellent cycle-to-cycle reproducibility and long-term operational stability. In addition, we performed stability measurements on back-gated MoS_2_ FETs without any encapsulation layer. Using the iso-current criterion at *I*_DS_ = 10 nA μm^–1^, the extracted threshold voltage remained highly stable over 100 consecutive cycles. The AIPS-gated MoS_2_ FET exhibited a mean *V*_th_ of approximately −3.3 ± 0.03 V, while the back-gated MoS_2_ FET showed a mean *V*_th_ of approximately 1.55 ± 0.025 V, demonstrating excellent cycle-to-cycle reproducibility and negligible threshold-voltage drift as shown in Supplementary Information 19b, further confirming the robustness of the MoS_2_ channel and device architecture under prolonged operation.

Furthermore, to examine the reversibility of the thermally activated ionic dynamics, we performed repeated thermal-cycling measurements on representative AIPS-gated MoS_2_ FETs under both dark and illuminated conditions. Specifically, dual-sweep top-gate transfer characteristics were measured at *T*_A_ = 25 °C and 75 °C over multiple thermal cycles, while maintaining *V*_BG_ = 2 V and *V*_DS_ = 1 V, as shown in Supplementary Information 20a-b. The *V*_Hyst_ was extracted using the iso-current criterion at *I*_DS_ = 10 nA μm^–1^. The extracted mean *V*_Hyst_ values are 1.62 ± 0.03 V (25 °C, dark), 2.08 ± 0.04 V (75 °C, dark), 1.47 ± 0.04 V (25 °C, light), and 1.95 ± 0.05 V (75 °C, light). The transfer curves remain nearly unchanged across successive cycles and consistently retrace the same hysteretic behavior. These results indicate that the ionic redistribution process is highly reversible and does not lead to cumulative degradation, irreversible ionic accumulation, or significant defect generation during repeated thermal operation. Finally, we also performed endurance measurements of the AIPS-gated MoS_2_ FETs over repeated electrical operation, as shown in Supplementary Information 21. Across electrical cycling from 10^0^ to 10^6^ cycles, the 2D FET exhibits a mean *V*_th_ of approximately −3.28 ± 0.03 V, demonstrating negligible threshold-voltage drift and excellent electrical endurance of the AIPS-gated MoS_2_ FET. This demonstrates excellent endurance of the dielectric/channel system under repeated gating. The excellent overlap of the transfer characteristics demonstrates the reliability and repeatability of the opto-ionic response.

### Thermally Regulated Photonic Fading Memory

Building on the established opto-ionic coupling in AIPS-gated MoS_2_ FETs, we exploit this effect to realize a thermally tunable photonic fading memory with relevance for brain-inspired computing. In neuromorphic frameworks, such as reservoir computing, devices are required to retain input information only for a finite duration before gradually forgetting it. This fading memory enables the encoding of temporal correlations in dynamic signals while preventing the accumulation of outdated states, allowing efficient processing of time-dependent information.

Time-resolved photocurrent measurements were performed under varying illumination intensities (*P*_in_ = 0.25–5 mW/cm^2^) and ambient temperatures (*T*_A_ = 0–75 °C) as shown in Figure 5a. At low *T*_A_, the device exhibits a high photoconductance ratio (*G*/*G*_0_) but with a slow rise time (*t*_rise_) and decay time (*t*_decay_), consistent with deep sub-bandgap trap occupancy and long-lived trapped charges. The persistent photoconductance (*G*_P_) reflects the retention of the photoinduced conductance state after illumination is removed and serves as a measure of the device’s memory persistence. At low temperatures, *G*_P_ decays slowly, indicating long-lived photoconductivity and a pronounced fading memory behavior. As the ambient temperature (*T*_A_) increases, the rise time (*t*_rise_) and decay time (*t*_decay_) progressively shorten, the photoconductive gain (*G*/*G*_0_) decreases, and *G*_P_ relaxes more rapidly after illumination is removed. This behavior signifies a transition from long-lived memory states to faster, transient responses governed by thermally activated ionic and trap-state dynamics: elevated temperatures enhance Ag^+^ mobility within AIPS and accelerate the release of trapped charges. This combination facilitates faster redistribution of ions and carrier recombination/detrapping, thereby reducing photoconductive gain but improving temporal resolution. Figure 5b-d present 3D bar plots of *G*/*G*_0_, *t*_rise_ and *t*_decay_ as functions of *P*_in_ and *T*_A_. Increasing *P*_in_ generally enhances *G*/*G*_0_ and accelerates the response, while temperature has a dual effect, enhancing response speed but diminishing photocurrent gain. Furthermore, the temporal evolution of *G*_P_ at different *T*_A_ values reveals progressively faster decay with increasing temperature. This temperature-dependent relaxation behavior directly enables thermally tunable fading memory, where the persistence of the photoinduced conductance state can be controlled through ionic and trap-mediated dynamics. Additionally, the temporal evolution of the photocurrent is highly reproducible across multiple illumination cycles, with consistent rise, peak, and decay characteristics as shown in Supplementary Information 22. The threshold voltage extracted at *I*_DS_ = 10 nA μm^–1^ remains highly reproducible over repeated measurements, with a mean value of −3.29 ± 0.02 V. The photoresponse also exhibits excellent cycle-to-cycle stability, with a mean peak photocurrent of 251 ± 12 nA, corresponding to a photocurrent amplitude of 204 ± 11 nA. The small cycle-to-cycle variations observed are within the expected experimental fluctuations and do not alter the overall fading memory behavior.

**Figure 5:**
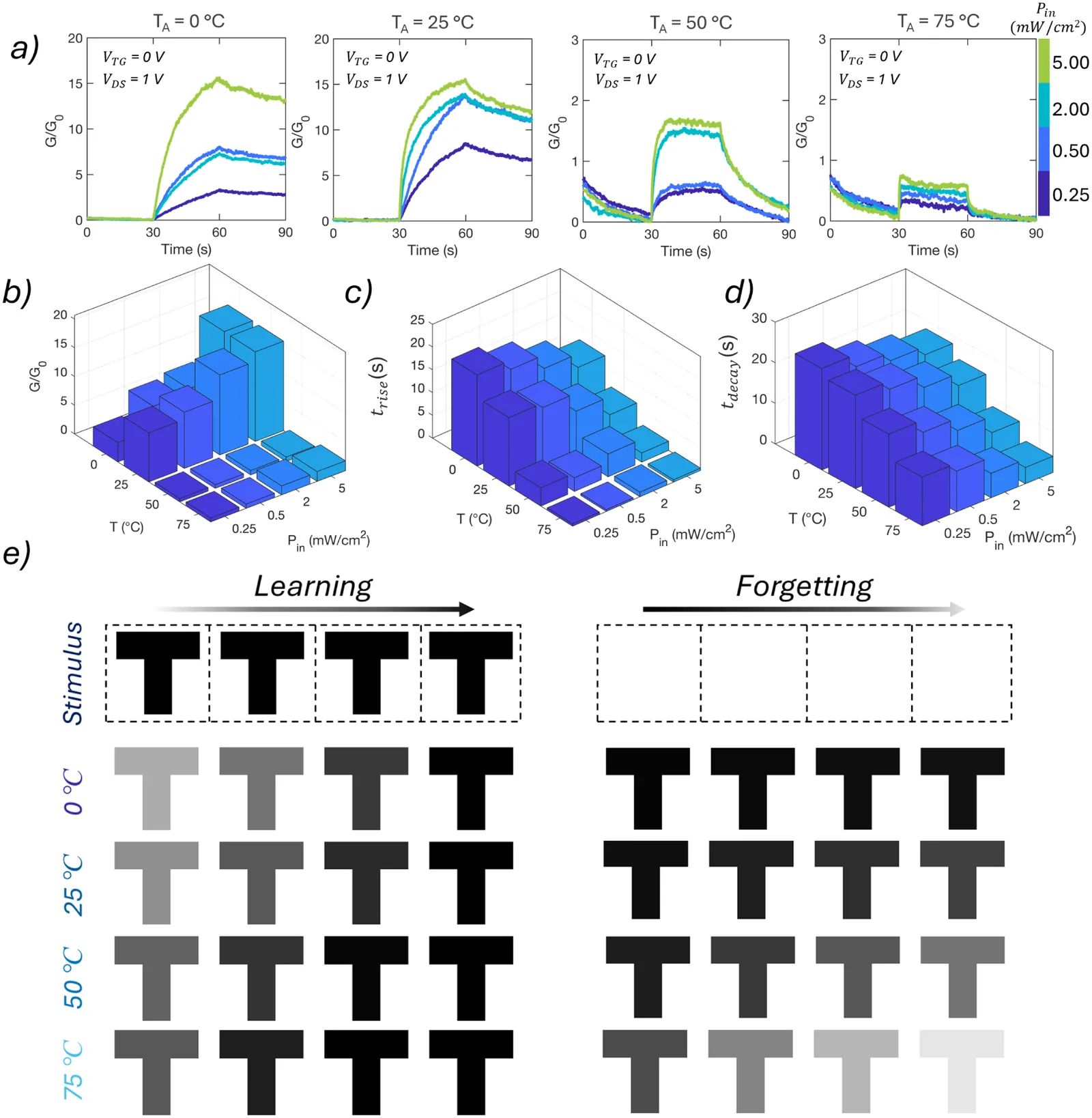
Temperature-mediated photonic fading memory in AIPS-gated MoS_2_ FETs. (a) Time-resolved photoresponse measured under varying illumination intensities (*P*_in_ = 0.25–5 mW cm^–2^) and ambient temperatures (*T*_A_ = 0–75 °C). At low *T*_A_, the device exhibits relatively higher photoconductive gain (*G*/*G*_0_) with slow rise (*t*_rise_) and decay (*t*_decay_) times, whereas increasing *T*_A_ accelerates the response and reduces persistence of the photoinduced conductance (*G_P_*). (b–d) 3D bar plots of *G*/*G*_0_, *t*_rise_, and *t*_decay_ as functions of *P*_in_ and *T*_A_. (e) Demonstration of photonic fading memory through encoding and recall of a 3 × 3-pixel letter “T”, with memory persistence tunable by temperature.

We also performed wavelength-dependent measurements to understand the origin of the observed opto-ionic modulation. All optical measurements were performed using RGB LEDs with wavelength ranges of blue (450–455 nm), green (495–570 nm), and red (625–750 nm), while the incident power density (*P*_in_) was varied from 0.14 to 10 mW cm^–2^. As shown in Supplementary Information 23, the photoresponses exhibit a strong wavelength dependence. The largest modulation is observed under blue illumination, followed by green illumination, whereas the response is strongly suppressed under red illumination. This trend indicates that efficient optical excitation of the AIPS dielectric is required to generate the observed conductance modulation. Notably, the strong suppression observed under red illumination is particularly important. While a weak residual response may arise from defect-mediated optical states, consistent with the measured AIPS photoluminescence peak near 1.95 eV, the substantially reduced modulation demonstrates that low-energy photons are significantly less effective at generating the charge redistribution required for interfacial electrostatic modulation. Consequently, the wavelength-dependent measurements support the conclusion that the observed behavior originates from photoexcitation within AIPS and its coupling to ionic and electronic processes at the AIPS/MoS_2_ interface, rather than from a purely photoconductive response of the MoS_2_ channel. More broadly, this behavior emerges from a coupled photonic-ionic-electronic interaction in the vdW AIPS/MoS_2_ heterostructure, where light absorption, lattice vibrations, ion motion, and carrier dynamics collectively determine the temporal evolution of the device state. Such multi-modal coupling provides a mechanism for engineering neuromorphic device functionalities in which light and temperature jointly regulate memory persistence and fading characteristics.

### Image Reconstruction Using Thermally Regulated Photonic Memory

Having established the temperature-regulated fading behavior of the photonic memory in AIPS-gated MoS_2_ FETs, we finally exploit this property to demonstrate image reconstruction. The underlying concept is that the *G*_P_ serves as an electrical proxy for pixel intensity, with its retention time tunable via *T*_A_. At lower temperatures, the high *G*_P_ ensures long-lived memory for each pixel, while elevated temperatures accelerate decay, mimicking short-term memory and eventually forgetting.

We designed a proof-of-concept experiment to demonstrate the concept of biologically inspired, context-aware photonic memory in AIPS-gated MoS_2_ FETs by encoding and recalling a simple 3 × 3-pixel image of the letter “T”. Each pixel is defined electrically, with a dark pixel corresponding to illumination at *P*_in_ = 5 mW/cm^2^ and a bright pixel corresponding to *P*_in_ = 0 mW/cm^2^. The image is “written” by sequentially programming the conductance state of the device for each pixel, and the conductance evolution during and after illumination is used to reconstruct the image in the electrical domain as shown in Figure 5e.

At low temperature *T*_A_, the dark pixels form gradually due to slow Ag^+^ ion migration and defect-state filling, but once established, the image persists for a long duration. This behavior mimics long-term memory retention in cold-adapted animals, where learned information is preserved to support survival under slow-changing conditions. At high temperature *T*_A_, dark pixels appear almost instantly because temperature-assisted ion migration and charge trapping occur rapidly, but the stored pattern fades within seconds to minutes, analogous to short-term memory in biological systems such as insects, which rely on rapid yet transient visual memory for quick responses. By simply changing the ambient temperature, the same physical device can switch between persistent storage and fast reset fading memory modes, without external reconfiguration. This versatility stems from the intertwined photonic-ionic-electronic coupling in the AIPS/MoS_2_ heterostructure, where light defines the pixel states, temperature regulates ion migration rates, and ionic motion dynamically alters the channel’s electrostatics. Such adaptive retention control could enable neuromorphic vision hardware that tailors its memory behavior to environmental conditions, retaining critical visual cues in stable, low-temperature settings while enabling rapid decision-making and memory clearance in high-temperature or high-activity scenarios.

## Conclusion

In conclusion, this work establishes a new design principle for transistor architectures in which the gate dielectric is no longer a passive electrostatic element but an active, multimodal interface that integrates sensing, memory, and logic functions through intrinsic multiphysics coupling. By exploiting the independent yet converging roles of defect-mediated photoactivity and temperature-assisted ionic migration in AIPS, we demonstrate how a vdW dielectric can directly encode environmental context into electronic function. This approach challenges the conventional separation of sensing and gating layers, offering a compact, materials-driven pathway to adaptive, energy-efficient electronics. Beyond revealing AIPS as a new high-κ vdW thiophosphate, our findings suggest a broader class of functional dielectrics in which photons, ions, and electrons are deliberately coupled, opening opportunities for context-aware devices in greenhouse monitoring, cold-chain logistics, autonomous edge nodes, and future neuromorphic systems.

## Methods

### Synthesis of AgInP_2_S_6_ Crystals

AgInP_2_S_6_ crystals were grown using the Chemical Vapor Transport (CVT) method in a two-zone furnace directly from the elements. Silver (chunks, 99.9%, Sigma-Aldrich, Czechia), Indium (99.99%, −100 mesh, Wuhan Xinrong New Materials Co., China), phosphorus (99.9999%, 2–6 mm granules, Wuhan Xinrong New Materials Co., China), and sulfur (99.9999%, 2–6 mm granules, Wuhan Xinrong New Materials Co., China) were placed in a stoichiometric ratio corresponding to 12 g of AgInP_2_S_6_ in a quartz ampoule (40 × 220 mm) together with 0.5 g of iodine (99.9%, Fisher Scientific, United Kingdom) and melt-sealed under high vacuum (pressure under 1 × 10^–3^ Pa using an oil diffusion pump with liquid nitrogen cold trap and liquid nitrogen cooling of the ampoule with an oxygen–hydrogen welding torch). The ampoule was placed horizontally in a muffle furnace and heated to 450 °C for 25 h (heating rate 1 °C/min) and subsequently to 500 °C for 50 h and to 600 °C for 50 h with a heating rate of 0.1 °C/min and a cooling rate of 1 °C/min. The prereacted AgInP_2_S_6_ was mechanically homogenized inside the ampoule and placed in a horizontal two-zone furnace. First, the source zone was heated to 600 °C and the growth zone to 750 °C for 2 days, and subsequently, the thermal gradient was reversed, and the source zone was heated on 700 °C and the growth zone on 600 °C for 10 days. Finally, after cooling, the growth zone was heated on 200 °C for 2 h while the source zone was kept around room temperature to remove excess of transport medium, and the ampoule was opened in an argon filled glovebox and the formed AgInP_2_S_6_ crystals were collected.

### Large-Area MoS_2_ Growth

Monolayer MoS_2_ was grown on epi-ready 2″ c-plane sapphire substrates via metal–organic chemical vapor deposition (MOCVD) in a cold-wall horizontal reactor equipped with an inductively heated graphite susceptor and wafer rotation to ensure uniform growth. Molybdenum hexacarbonyl (Mo­(CO)_6_) and hydrogen sulfide (H_2_S) were used as precursors. The Mo­(CO)_6_ source was maintained at 25 °C and 375 Torr in a stainless-steel bubbler, delivering 2.0 × 10^–2^ sccm of metal precursor, while 400 sccm of H_2_S was supplied during growth. Deposition was carried out at 950 °C and 50 Torr in an H_2_ ambient, with monolayer formation achieved within 18 min. Prior to growth, the substrate was annealed at 1000 °C in H_2_ for 10 min to improve surface quality. After growth, the sample was cooled under H_2_S flow to 300 °C to suppress film decomposition.

### Raman and Photoluminescence Spectroscopy

Raman spectroscopy of the AgInP_2_S_6_ crystals was performed using a Horiba LabRAM HR Evolution confocal Raman microscope with a 532 nm laser and a grating spacing of 1800 gr/mm for Raman spectroscopy and 300 gr/mm for PL spectroscopy.

### X-ray Diffraction

Powder X-ray diffraction patterns of synthesized crystals were obtained using a Rigaku MiniFlex 600 X-ray diffraction system equipped with a D/teX silicon 1D detector. The Cu Kα radiation (wavelength λ = 1.5406 Å) was generated by applying a voltage of 40 kV and a current of 15 mA. The radiation was filtered with a graphite monochromator and a Kβ foil filter. A zero-background silicon holder was used for the measurements.

### X-ray Photoelectron Spectroscopy

X-ray photoelectron spectroscopy (XPS) measurements were performed using a Thermo Scientific NEXSA G2 XPS instrument equipped with an electron flood gun and a scanning ion gun at room temperature. The crystals were placed on copper tape, which served as an adhesive substrate. The data were analyzed using the Thermo Scientific Avantage Data System software.

### Atomic Force Microscopy

Atomic force microscopy was used to determine the thickness and surface morphology of exfoliated AIPS flakes. RTESPA-150 probe tips were used with a Bruker Dimension Icon AFM. All images were collected in PeakForce Tapping mode with a peak force of 12 nN and a scan rate of 0.5 Hz. Images were processed and exported by using NanoScope.

### Scanning Electron Microscopy

Scanning electron microscopy (SEM) of the 2D MoS_2_ transistors used in this study was conducted using a Zeiss Gemini 500 field-emission scanning electron microscopy (FESEM) system at an accelerating voltage of 5 kV.

### Fabrication of Dual-Gated 2D FETs

To begin with, monolayer MoS_2_ was transferred on 25 nm thick Al_2_O_3_ substrates. The substrate was spin-coated with PMMA A6 (4000 rpm for 45 s) and baked at 180 °C for 90 s. The resist was then exposed using a Raith EBPG5200 e-beam lithography tool and developed with a 1:1 mixture of 4-methyl-2-pentanone (MIBK) and IPA for 60 s, followed by a 45 s IPA rinse. The exposed monolayer MoS_2_ film was etched using an SF_6_ RIE process at 5 °C for 30 s. Afterward, the sample was cleaned in acetone and IPA to remove the e-beam resist. Next, the 2D dielectrics were mechanically exfoliated on top of the prepatterned MoS_2_ channels. A second lithography step was performed to form the source/drain and top-gate electrodes. The substrate was spin-coated at 4000 rpm for 45 s with methyl methacrylate (MMA) EL6 and PMMA A3; these resists were baked at 150 °C and 180 °C for 90 s each. E-beam lithography was used again to pattern the source and drain, followed by development with MIBK/IPA and an IPA rinse, as before. For MoS_2_ FETs, 20 nm of Ni and 60 nm of Au were deposited via e-beam evaporation to form the electrodes. Finally, a liftoff process was conducted to remove excess Ni/Au by soaking the sample in acetone for 1 h, followed by a 10 min IPA rinse to clean the substrate.

### Electrical Characterization

Electrical characterization of the fabricated devices was performed using a semiautomated FormFactor 11000 probe station under atmospheric conditions with a Keysight B1500A parameter analyzer. A continuous-wave white light source was used for all experiments involving light illumination unless otherwise stated.

## Supplementary Material



## Data Availability

Data sets generated during and/or analyzed during the current study are available from the corresponding author on reasonable request. The codes used for plotting the data are available from the corresponding authors on reasonable request.

## Notes

AFM thickness measurements and photoluminescence spectroscopy of AIPS flakes; electrical characteristics, electrostatic coupling, gate leakage, and hysteresis of AIPS-gated MoS_2_ FETs; defect-mediated photoexcitation, AIPS/MoS_2_ band alignment, and DFT-based defect analysis; control photoresponse measurements in back-gated MoS_2_ FETs; temperature-, illumination-, and sweep-time-dependent opto-ionic dynamics; Arrhenius analysis of effective Ag^+^ migration barriers; virtual-source and drift–diffusion modeling of Ag^+^ redistribution, ionic mobility, and diffusivity; long-term operational stability, thermal reversibility, and electrical endurance up to 10^6^ cycles; cycle-to-cycle reproducibility of electrical and photoresponse characteristics; and wavelength-dependent photoresponse under blue, green, and red LED illumination (PDF)
