# Supplementary material for: Thermally Tunable Fading Memory Enabled by Opto-Ionic-Electronic Coupling in AgInP2S6‑Gated Two-Dimensional Field-Effect Transistors
Source: ACS Nano. 2026 Oct 5;20(41):27665–77. doi: 10.1021/acsnano.5c21149 (PMC13689912; doi:10.1021/acsnano.5c21149)
Supplement: Supplementary file 1 [file nn5c21149_si_001.pdf]

Supplementary Information

Thermally tunable fading memory enabled by opto-ionic-electronic coupling in $AgInP_2S_6$-gated two-dimensional field effect transistors

Dipanjan Sen[1], Anirban Chowdhury[1], Anshul Rasyotra[1], Lei Ding[1], Rui Gusmao[2], Subir Ghosh[1], Arpan Ghosh[1], Joan M Redwing[3,4], Divya Somvanshi[5], Yang Yang[1], Zdenek Sofer[2], and Saptarshi Das[1,4,6]*

[1]Engineering Science and Mechanics, Penn State University, University Park, PA 16802, USA

[2]Department of Inorganic Chemistry, University of Chemistry and Technology Prague, Prague 16000, Czech Republic

[3]2DCC, Penn State University, University Park, PA 16802, USA

[4]Materials Science and Engineering, Penn State University, University Park, PA 16802, USA

[5] Department of Physics, Harcourt Butler Technical University, Kanpur, Uttar Pradesh 208002, India

[6]Electrical Engineering, Penn State University, University Park, PA 16802, USA

[*] Corresponding Author

Supplementary Information 1

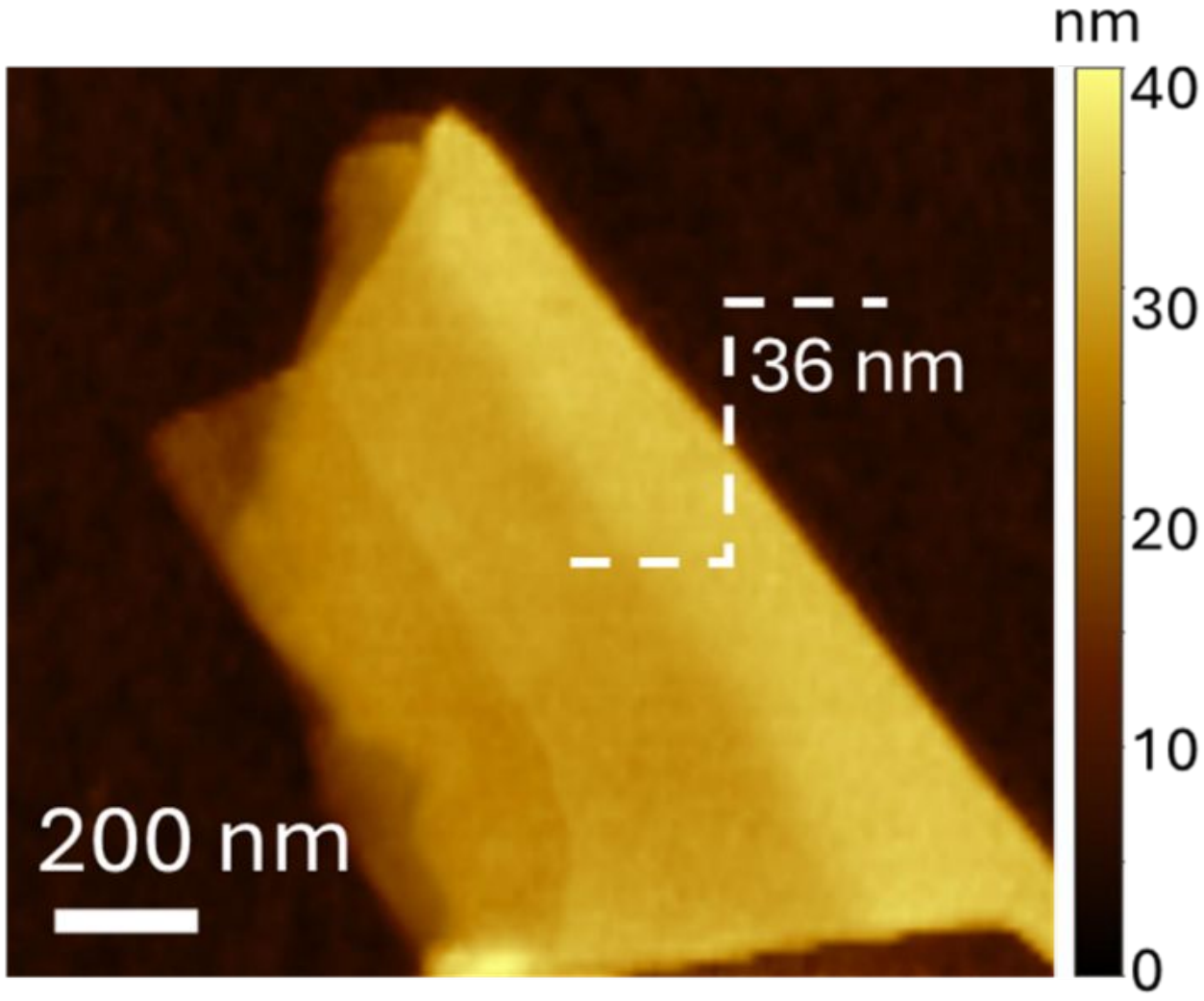

Supplementary Information. 1. *AFM image of an exfoliated AIPS flake with a uniform thickness of 36 nm.*

Supplementary Information 2

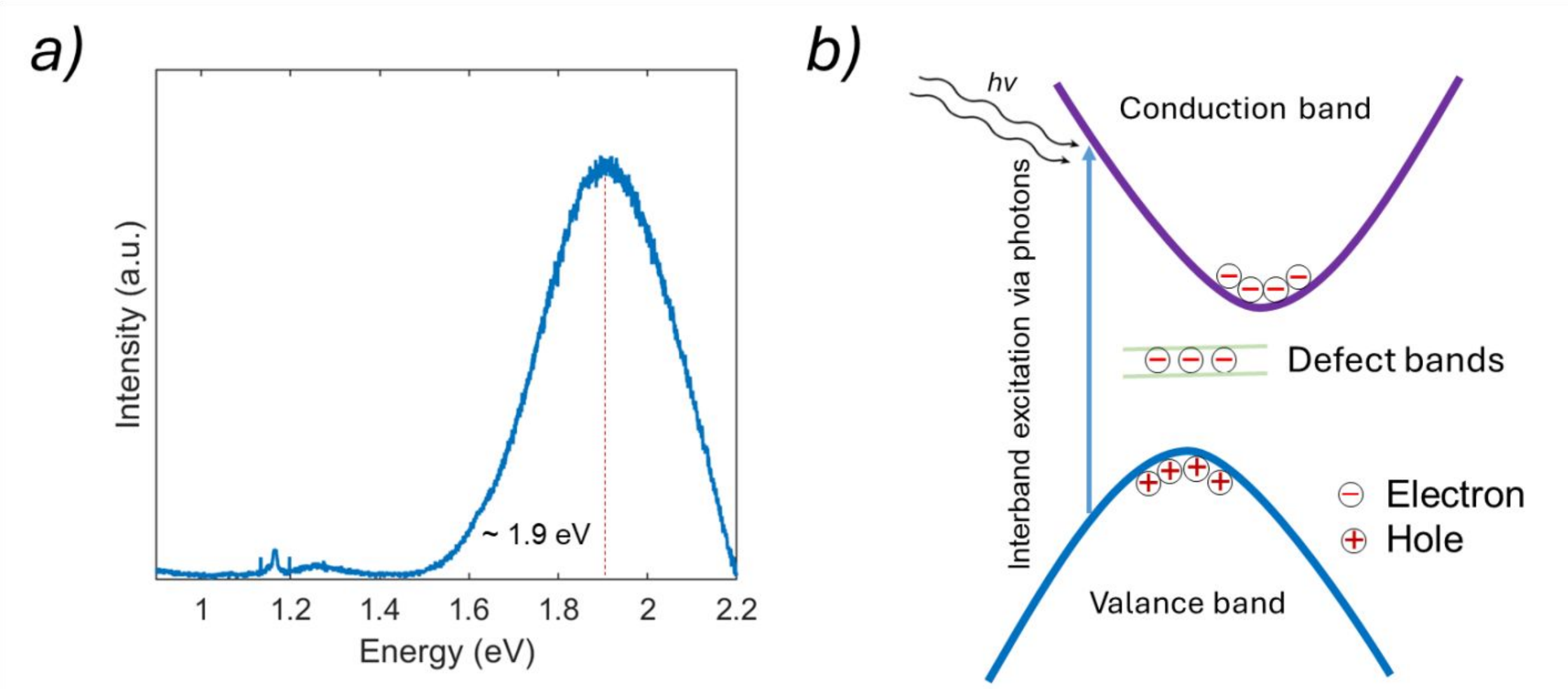

Supplementary Information 2. Photoluminescence spectroscopy and defect-mediated photoexcitation in AIPS. *(a) Photoluminescence spectrum with an emission peak near 1.95 eV and (b) band diagram showing inter band excitation and defect-assisted trapping.*

Supplementary Information 3

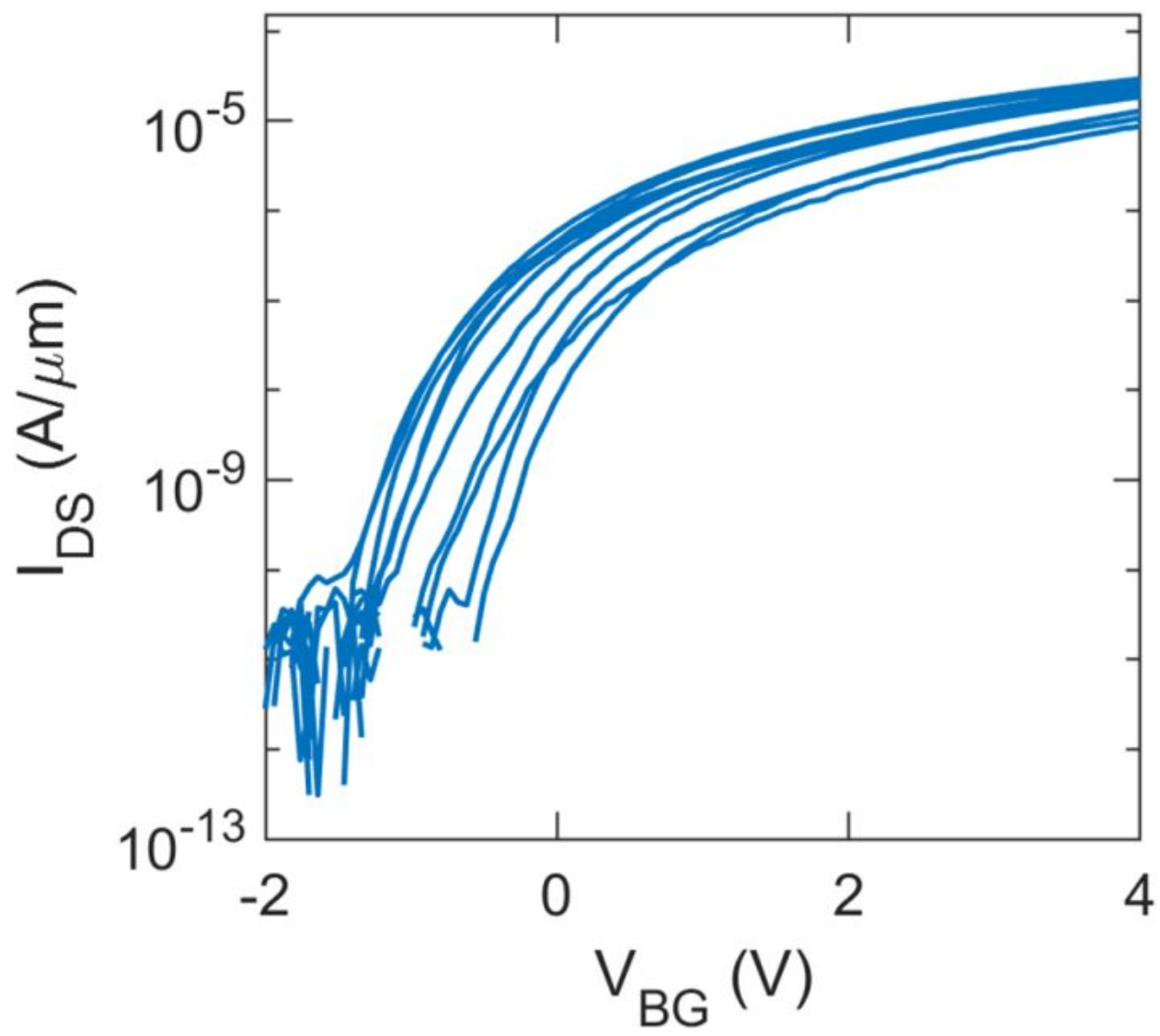

Supplementary Information 3. Back-gated MoS_2 FETs. *Transfer characteristics of 10 representative MoS_2 FETs measured by sweeping the back-gate voltage (V_{BG}) from 4 V to –2 V at a constant drain bias (V_{DS} = 1 V).*

Supplementary Information 4

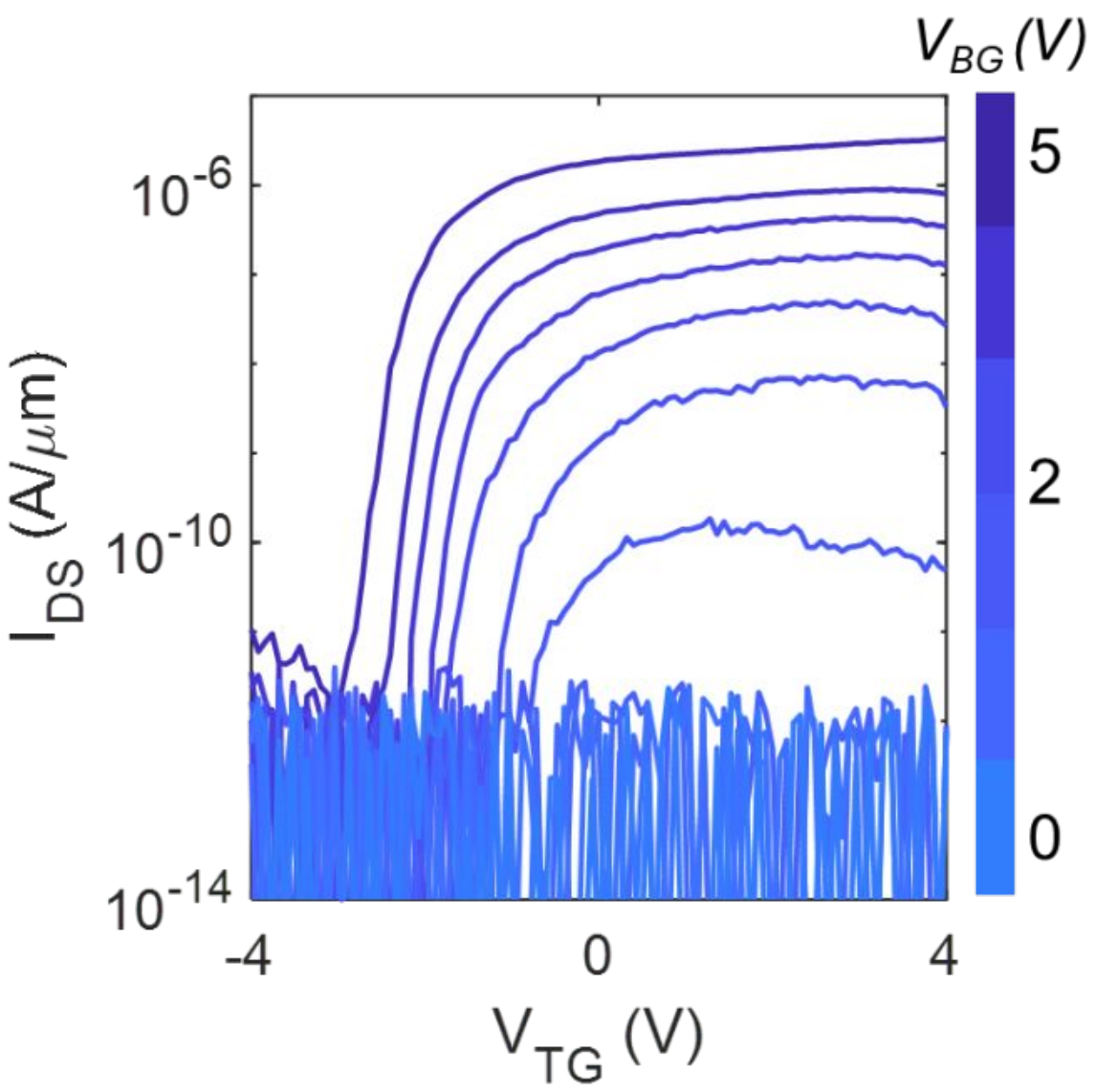

Supplementary Information 4. Electrostatic strength of AIPS-gated MoS_2 FET. *The top-gate transfer curve of a representative MoS_2 FET with AIPS dielectric was obtained by sweeping the V_{TG} from 4 V to -4 V for various V_{BG} values at a constant V_{DS} =1 V.*

Supplementary Information 5

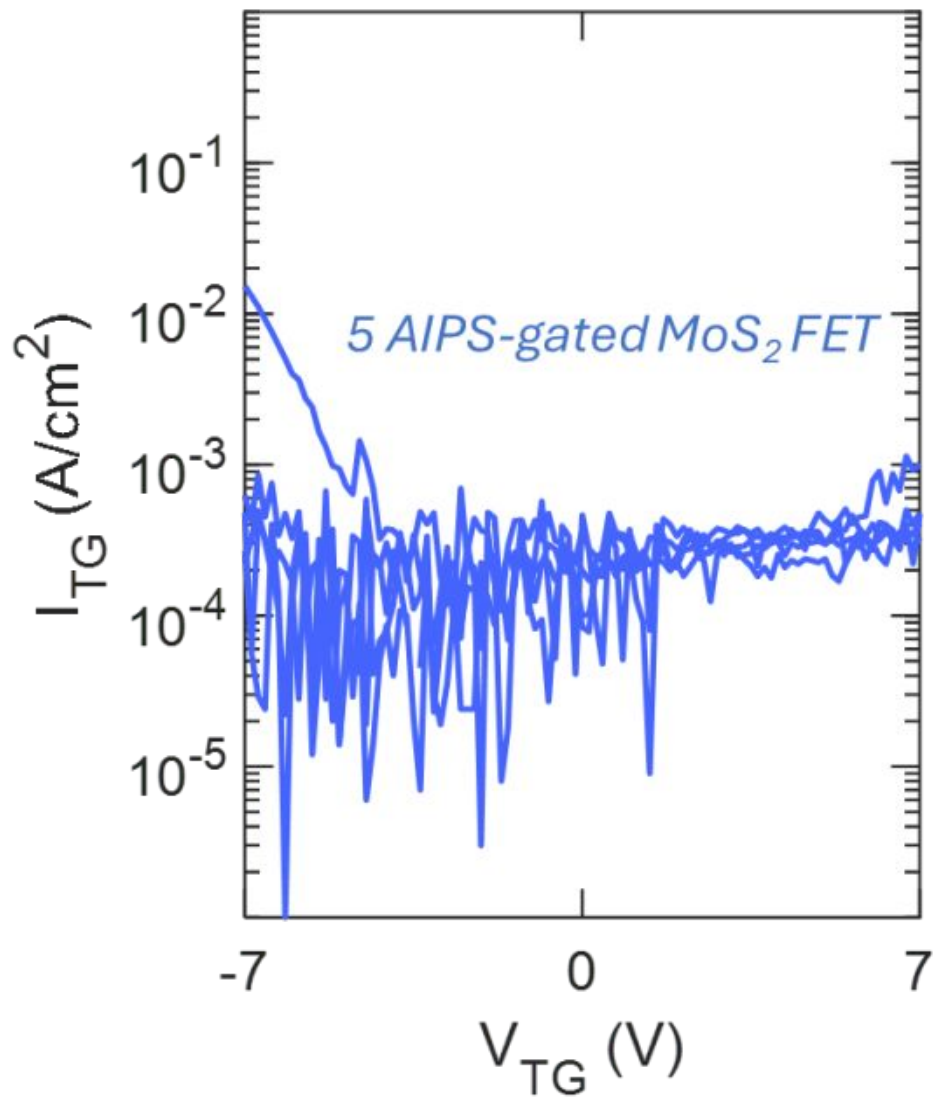

Supplementary Information 5. Gate leakage current in AIPS-gated MoS_2 FETs. *Gate leakage characteristics of 5 MoS_2 FETs gated with AIPS, measured by sweeping the top-gate voltage (V_{TG}) from –7 V to 7 V under a constant back-gate bias (V_{BG} = 4 V) and drain bias (V_{DS} = 1 V).*

Supplementary Information 6

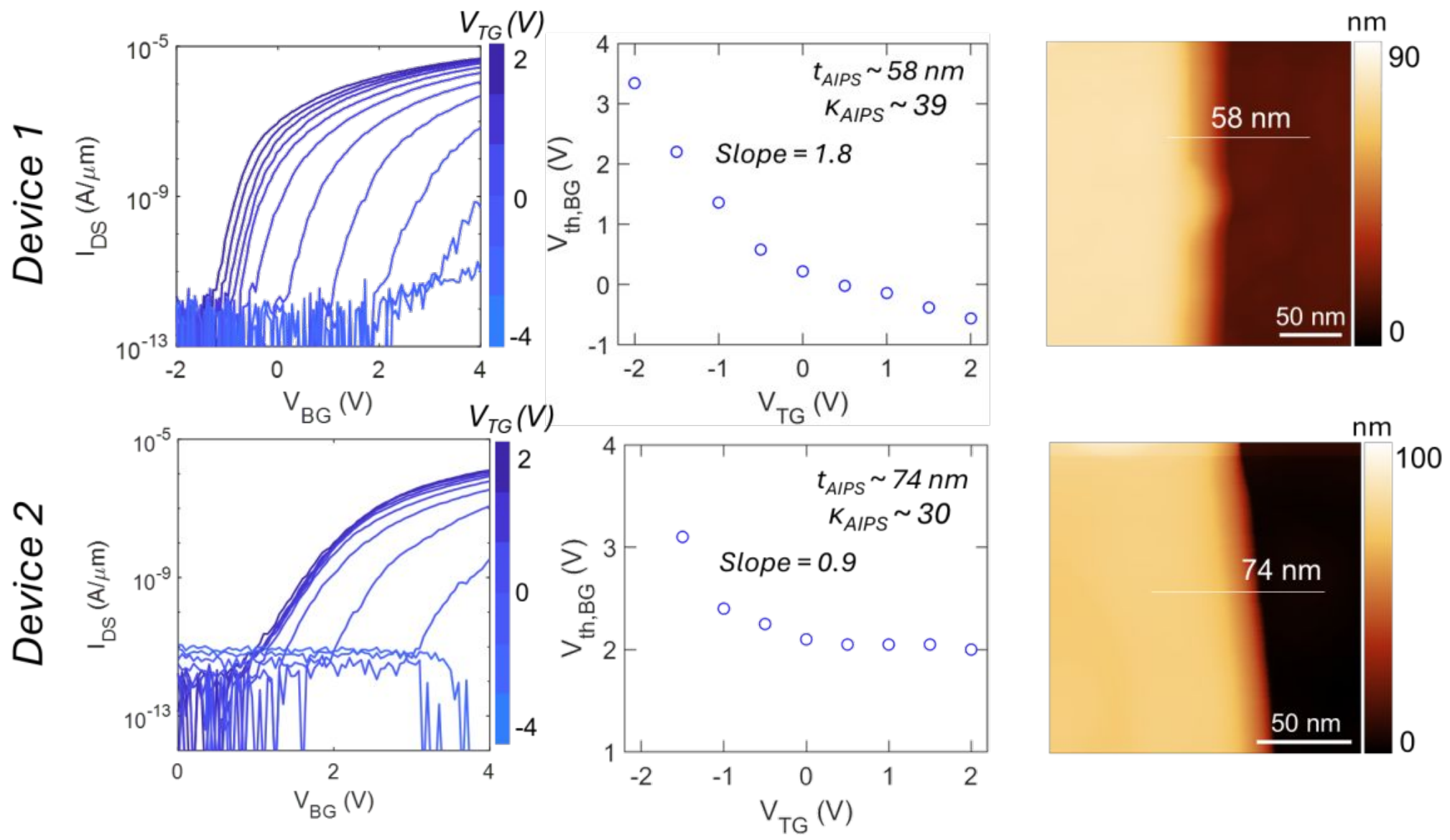

Supplementary Information 6. Dielectric strength of AIPS-gated MoS_2 FETs. *Back-gate transfer characteristics of two representative MoS_2 FETs with AIPS as the top-gate dielectric are shown alongside the extracted back-gate threshold voltage ($V_{th,BG}$) plotted as a function of V_{TG}. The slopes of $V_{th,BG}$ versus V_{TG} correspond to the ratio of the back-gate and top-gate EOTs, yielding effective dielectric constants of ~39 for a 58 nm-thick AIPS flake and ~30 for a 74 nm-thick AIPS flake, as confirmed by AFM thickness measurements.*

Supplementary Information 7

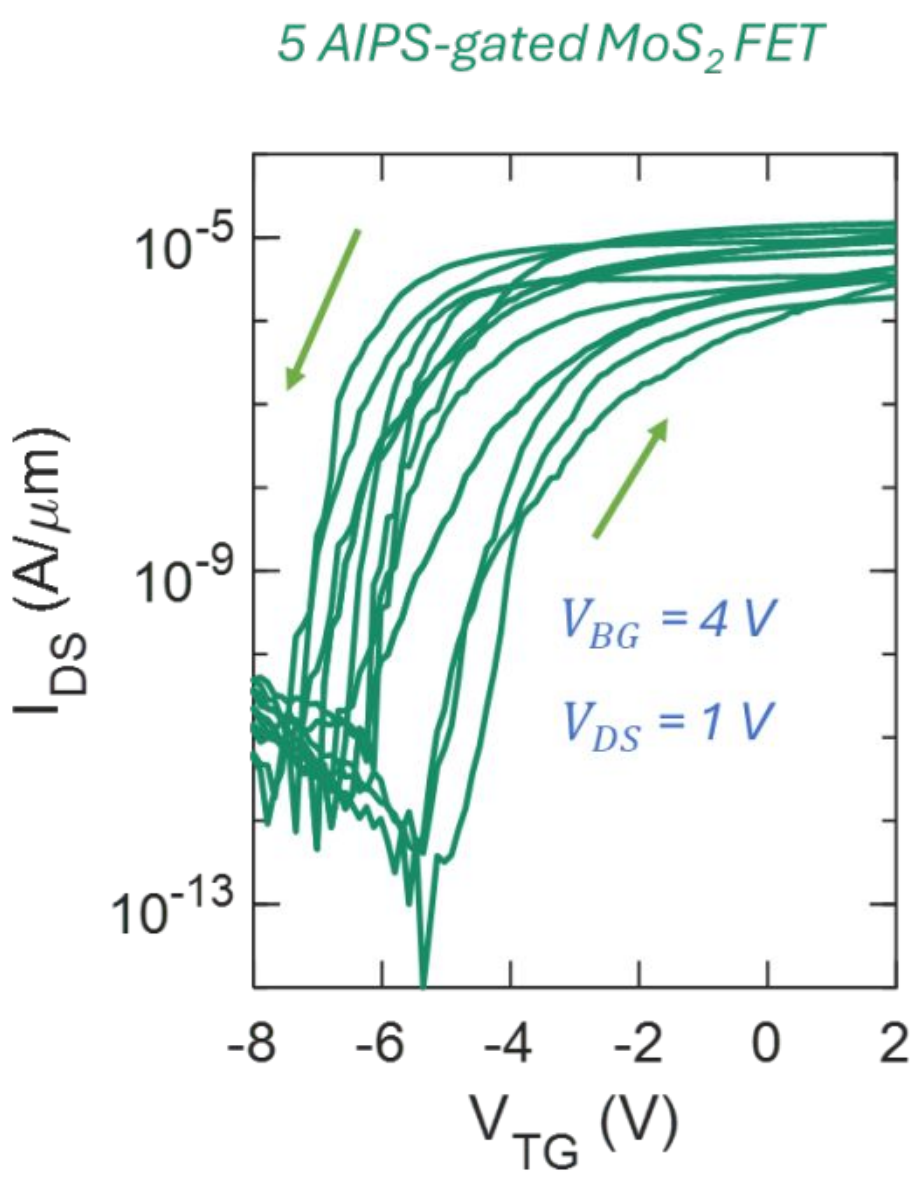

Supplementary Information 7. CCW hysteresis in multiple AIPS-gated MoS_2 FETs. *Dual-sweep top-gate transfer characteristic of 5 MoS_2 FETs gated with AIPS obtained by sweeping the V_{TG} from -8 V to 2 V at a constant V_{BG} = 4 V and V_{DS} = 1 V.*

Supplementary Information 8

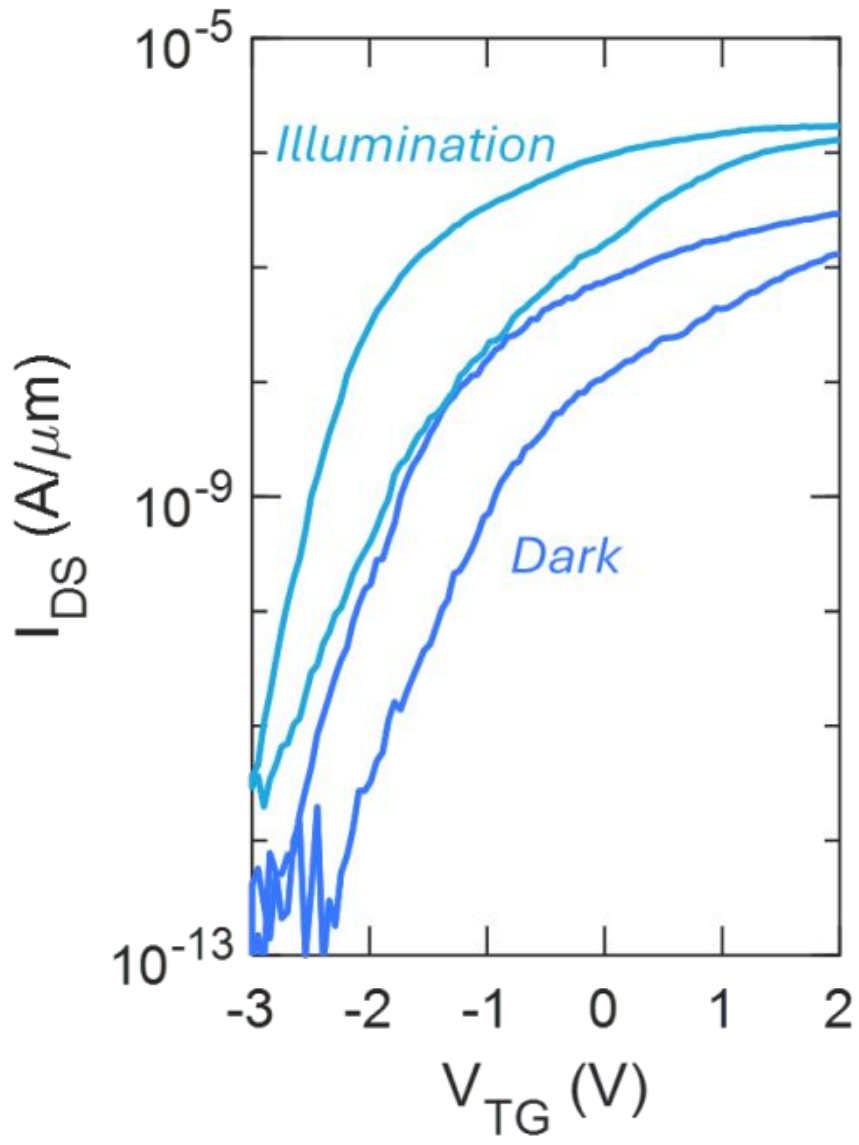

Supplementary Information 8. Defect-mediated photoactivity in AIPS-gated MoS_2 FET. *Dual-sweep top-gate transfer characteristics of AIPS-gated MoS_2 FET under dark and illumination (P_{in} = 5 mW/cm^2). Note that under illumination on-current (I_{on}) increases with a negative shift in $V_{th,TG}$ and decrease in V_{Hyst}.*

Supplementary Information 9

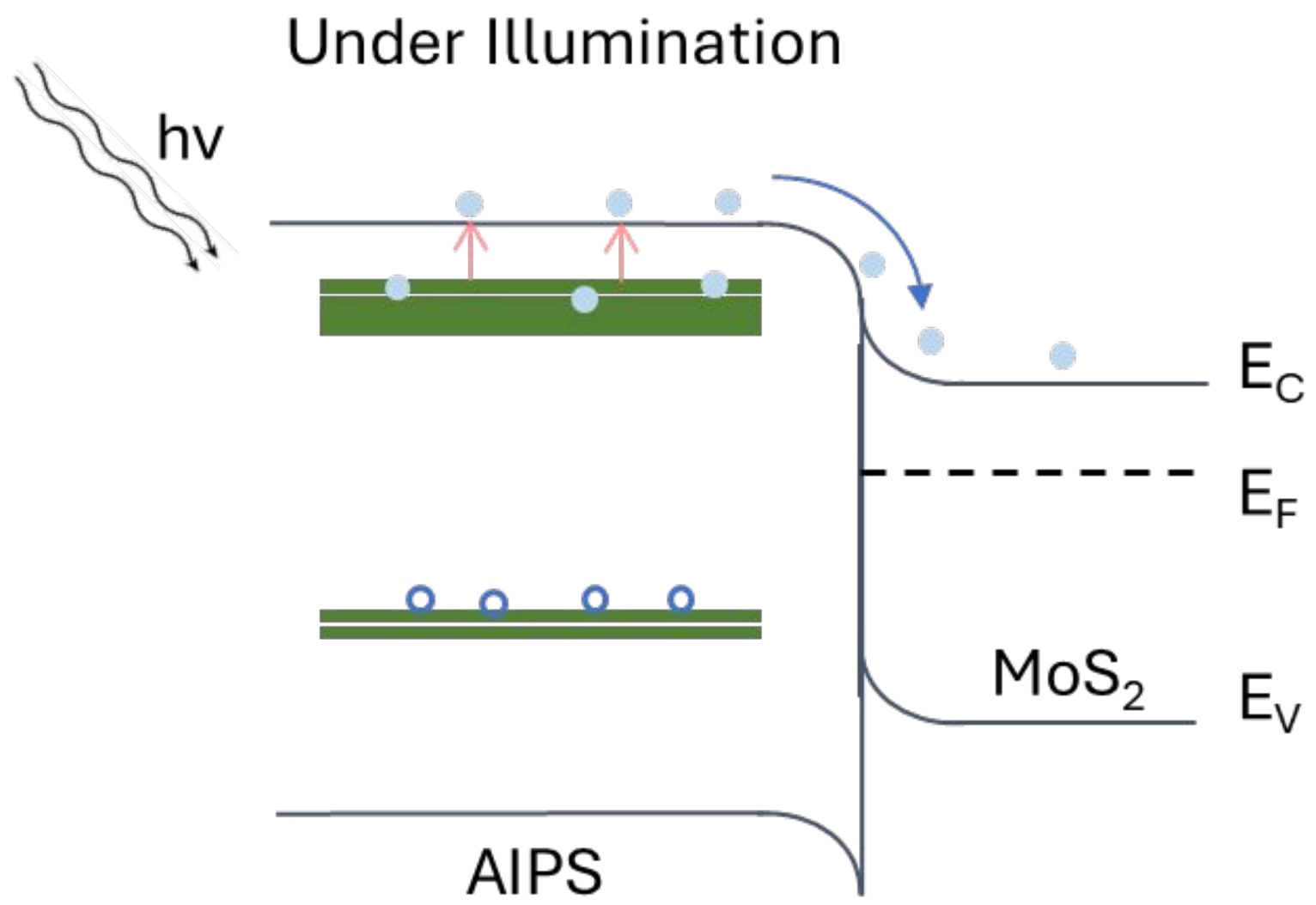

Supplementary Information 9. Band alignment of the AIPS-MoS_2 interface. *Sub-bandgap defect states in AIPS near the conduction band align close to the MoS_2 conduction band minima. This allows easy transfer of electrons into MoS_2 conduction band upon illumination.*

Supplementary Information 10

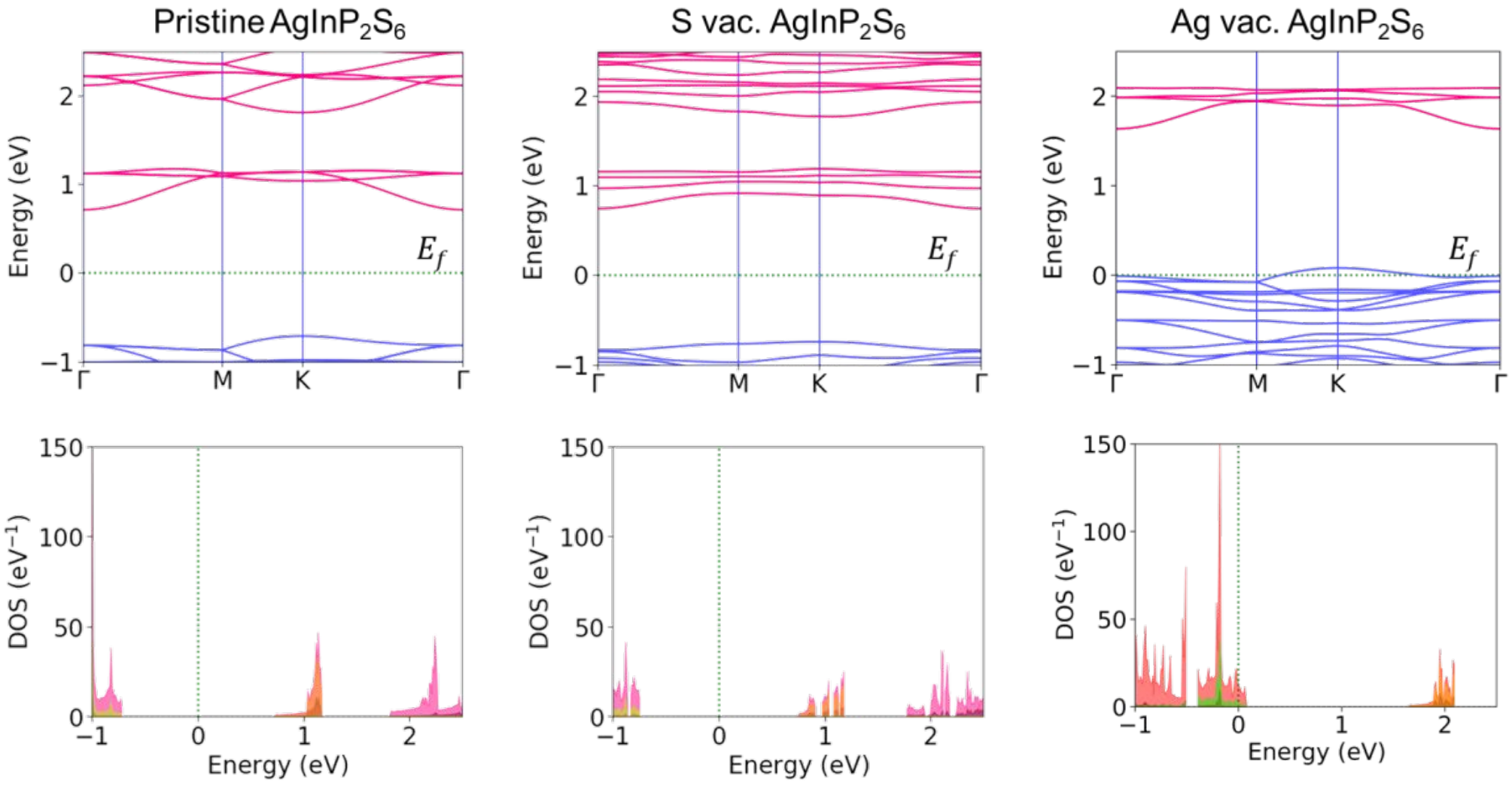

Supplementary Information 10. Band-structure analysis of different defect sites in AIPS. *Band structures and density of states exhibiting possible defect sites in AIPS consisting of S vacancies, and/or Ag vacancies, calculated through DFT simulations.*

Supplementary Information 11

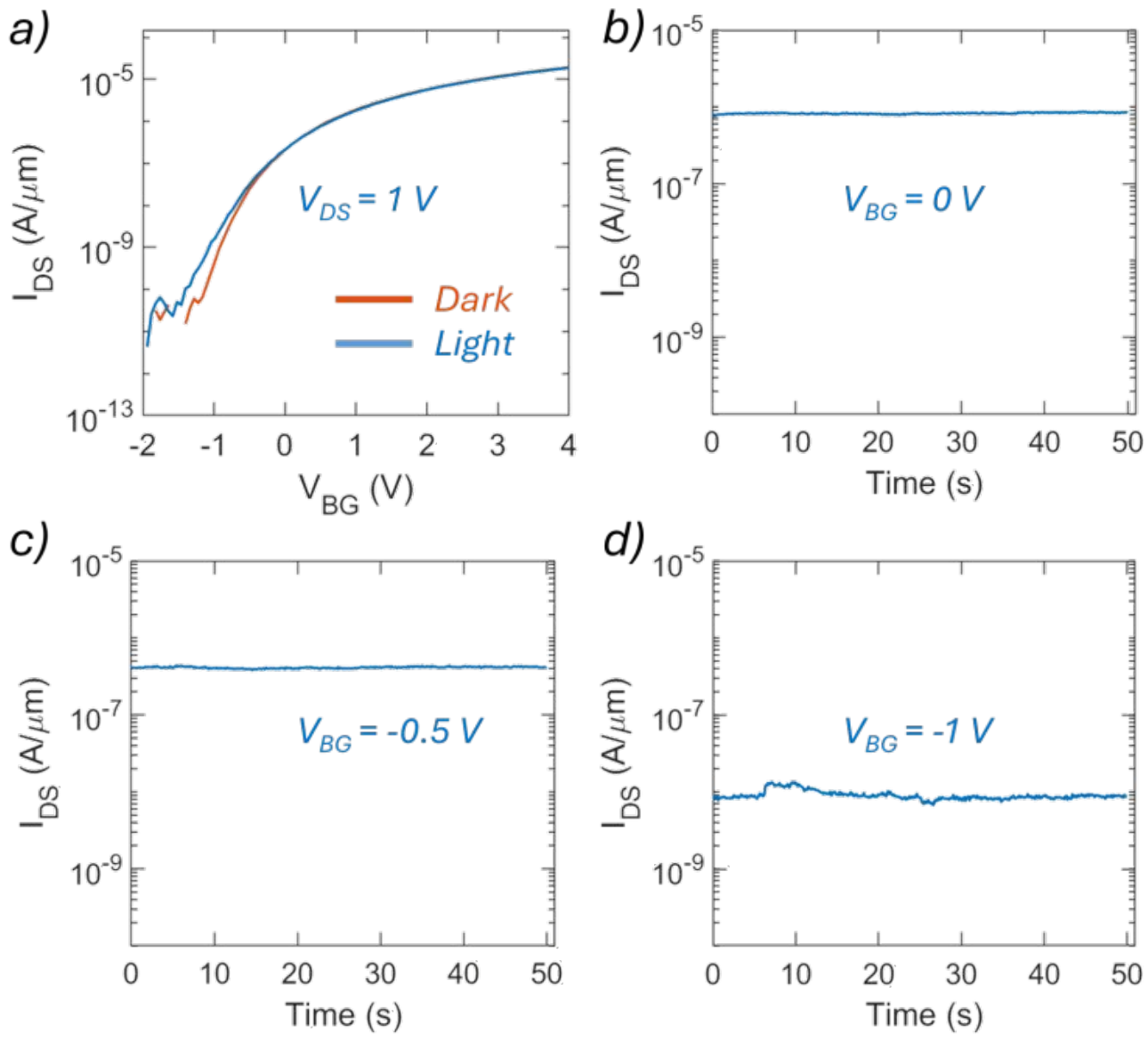

Supplementary Information 11. Photo-response in back-gated MoS_2 FETs. *(a) Back-gate transfer characteristics in dark and under illumination at* V_{DS} *= 1 V, showing negligible photoresponse under identical illumination, consistent with the weak optical absorption of monolayer* MoS_2*. (b–d) Time-resolved photo response under identical optical excitation at* V_{BG} *= 0 V, – 0.5 V, and –1 V.*

Supplementary Information 12

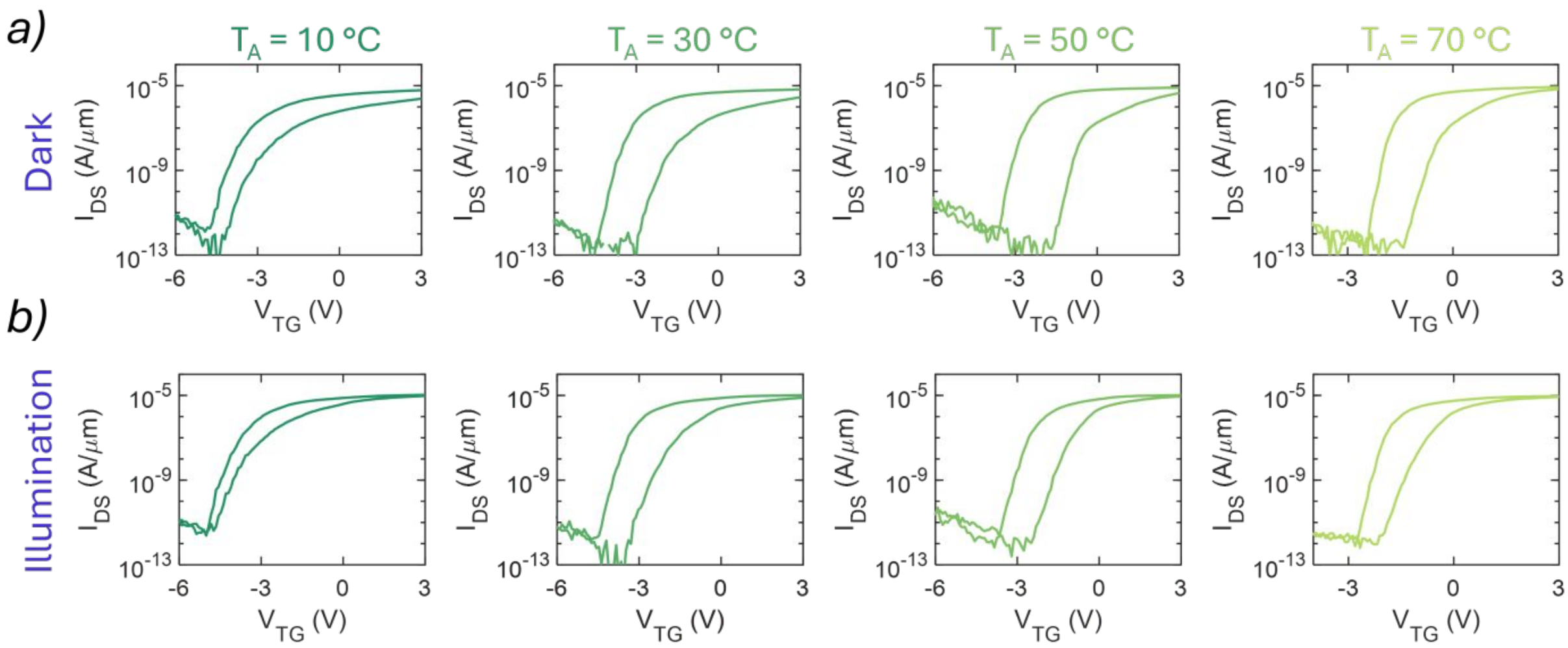

Supplementary Information 12. Opto-ionic coupling in AIPS-gated MoS_2 FETs. *Top-gate transfer curves of MoS_2 FETs recorded at different ambient temperatures (T_A =10 – 70 °C) under (a) dark and (b) illumination (P_{in} = 5 mW/cm²).*

Supplementary Information 13

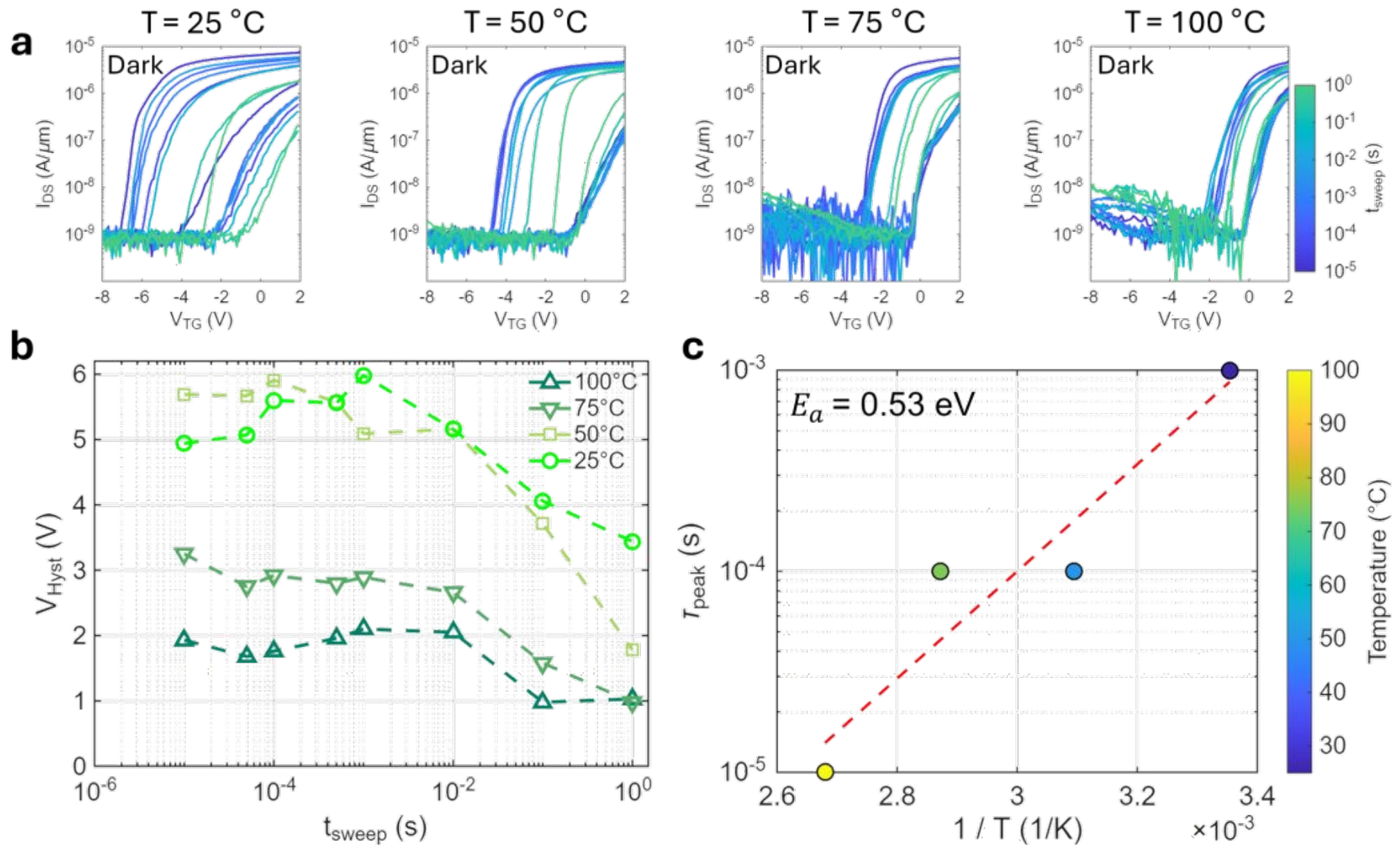

Supplementary Information 13. Sweep-time dependent Ag^+ migration dynamics in AIPS-gated MoS_2 FETs under dark. *(a) Dual-sweep top-gate characteristics of a representative AIPS-gated MoS_2 FET with t_{sweep} ranging between 10 μs to 1 s, measured at different temperatures with V_{BG} of 4 V. (b) Extracted hysteresis window (V_{Hyst}) plotted as a function of t_{sweep} for different temperatures. As temperature increases, t_{peak} shifts to lower values, reflecting thermally activated ion mobility, while the peak magnitude (V_{Hyst}) showing minimal change. (c) Arrhenius plot of the temperature-induced shift in V_{Hyst}, where the slope directly yields the effective migration barrier of Ag^+ ions. Notably, an effective migration barrier of E_a = 0.53 eV was extracted.*

Supplementary Information 14

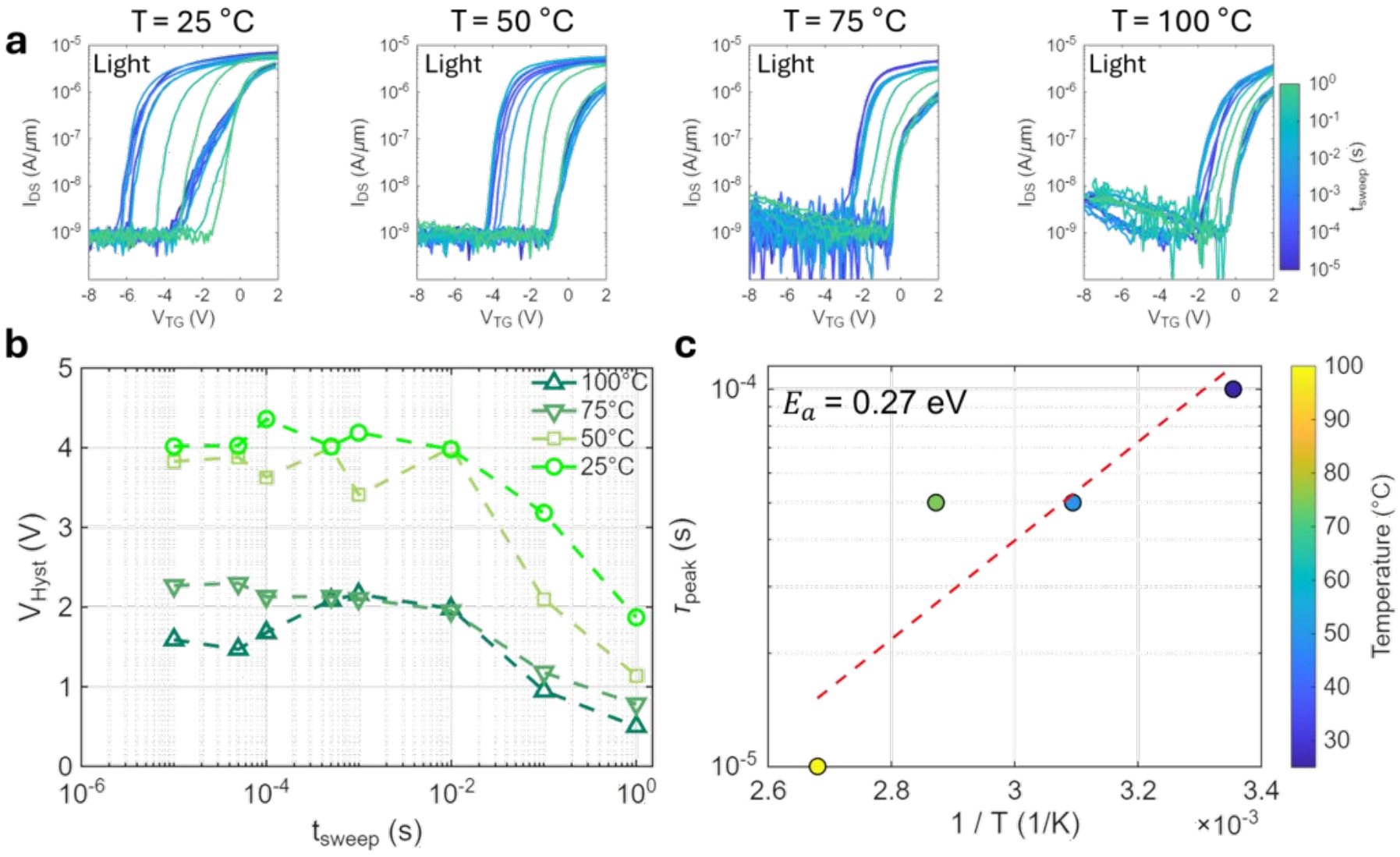

Supplementary Information 14. Sweep-time dependent Ag^+ migration dynamics in AIPS-gated MoS_2 FETs under light. *(a) Dual-sweep top-gate characteristics of a representative AIPS-gated MoS_2 FET with t_{sweep} ranging between 10 µs to 1 s, measured at different temperatures with V_{BG} of 4 V. (b) Extracted hysteresis window (V_{Hyst}) plotted as a function of t_{sweep} for different temperatures. As temperature increases, t_{peak} shifts to lower values, reflecting thermally activated ion mobility, while the peak magnitude (V_{Hyst}) showing minimal change. (c) Arrhenius plot of the temperature-induced shift in V_{Hyst}, where the slope directly yields the effective migration barrier of Ag^+ ions. Notably, an effective migration barrier of E_a = 0.27 eV was extracted.*

Supplementary Information 15

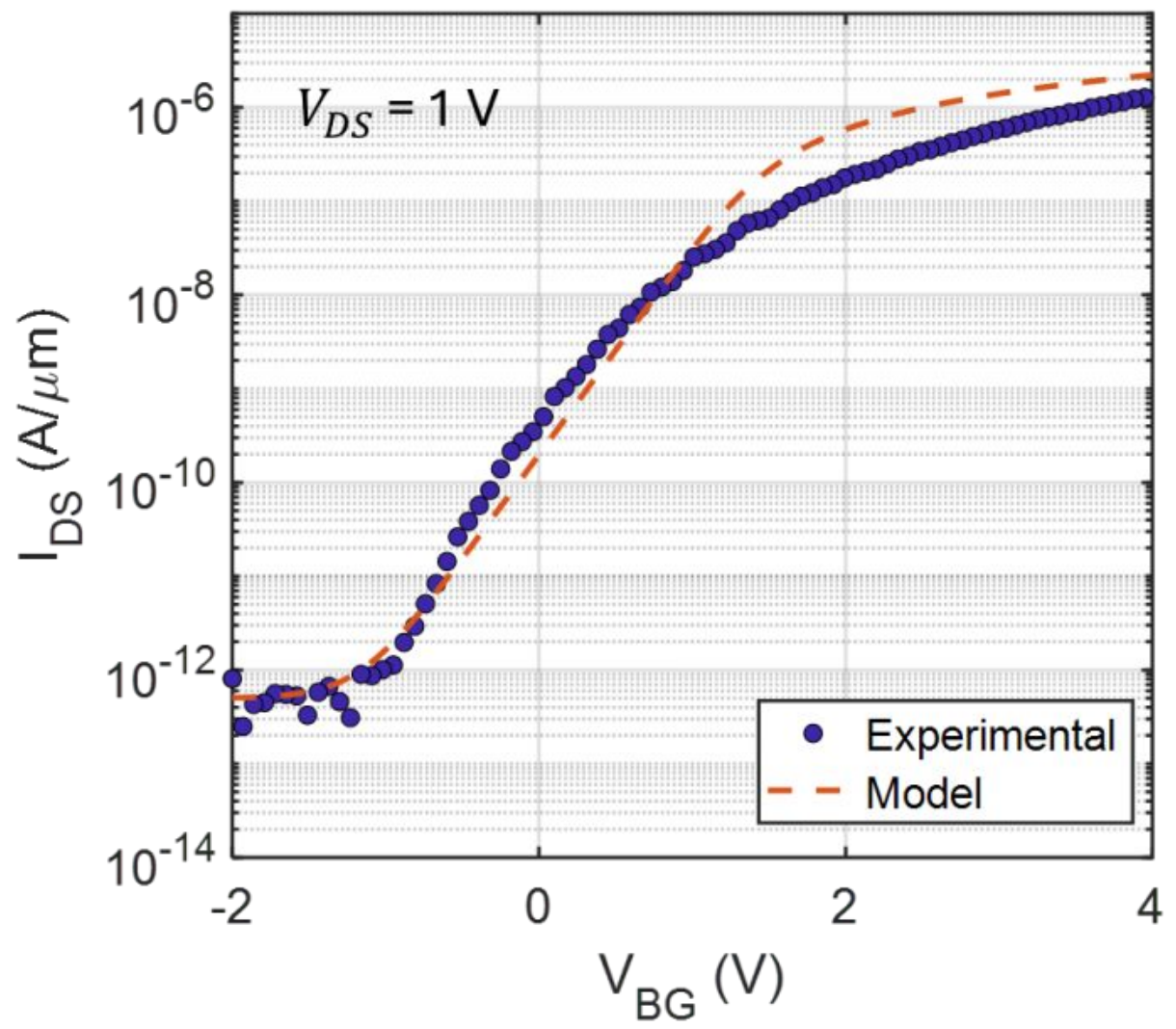

Supplementary Information 15. Virtual-Source Model for MoS_2 FETs. *Back-gate transfer characteristics of representative MoS_2 FETs with AIPS, as the top-gate dielectrics were measured by sweeping V_{BG} at V_{DS} =1 V alongside a theoretical fit obtained using a virtual-source model.*

Supplementary Information 16

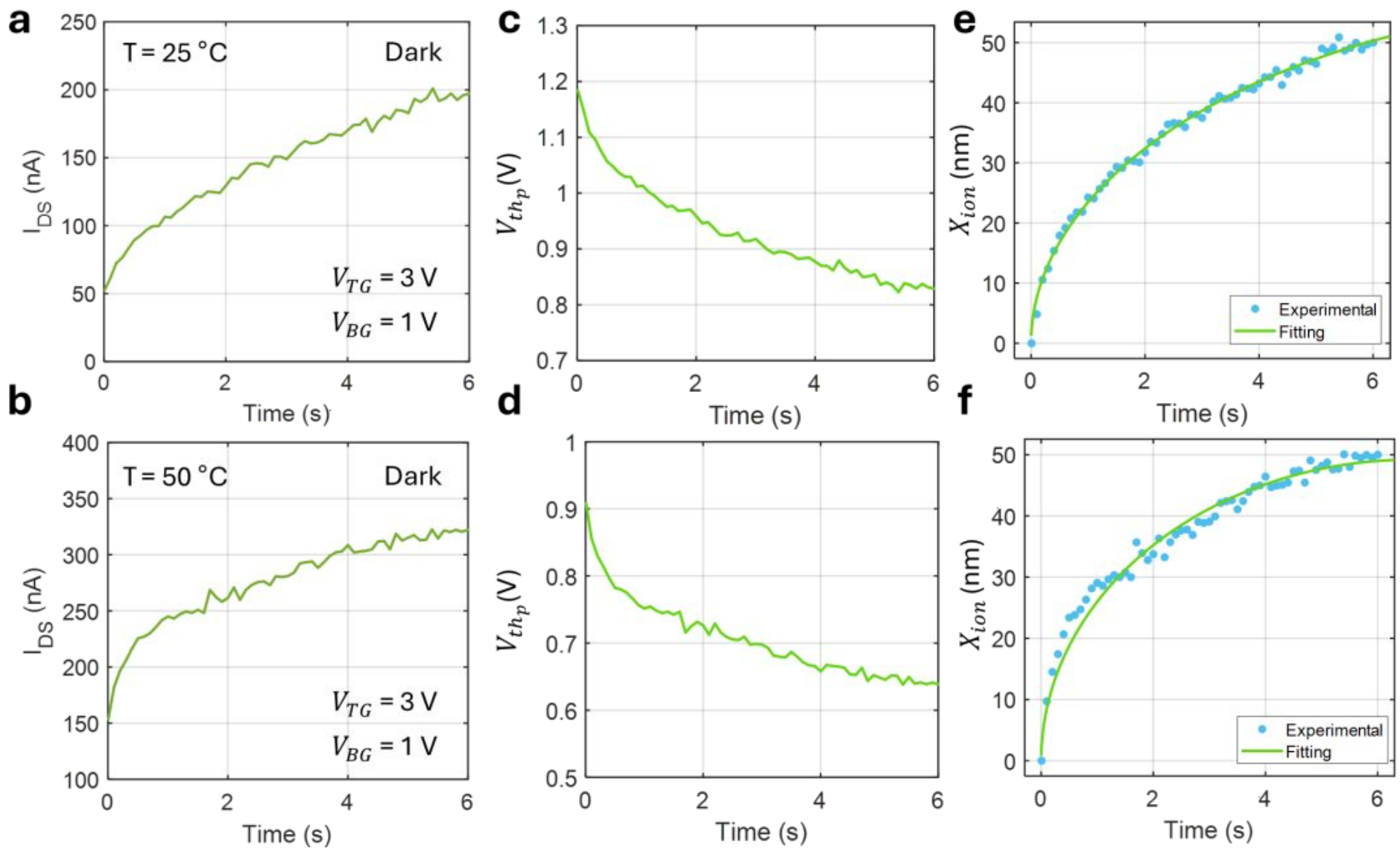

Supplementary Information 16. Virtual-Source Model for assessing V_{th} shift in AIPS-gated 2D FETs under dark. *(a-b) source-to-drain current (I_{DS}) of a MoS_2 FET as a function of time, for a constant top-gate voltage (V_{TG}) of 3 V and V_{DS} = 1 V, measured across different temperatures. Over time, the migration of Ag^+ ions within AIPS gradually shifts V_{th_p}, leading to a continuous and monotonic rise in I_{DS}. (c-d) Extracted V_{th_p} evolution over time exhibits a steady, monotonic decrease. Notably, the change in V_{th_p} is accelerated at higher temperatures, attributed to the enhanced ionic mobility. (e-f) Evolution of the Ag^+ ion charge distribution centroid, X_{ion}, over time, presented alongside a theoretical fit.*

Supplementary Information 17

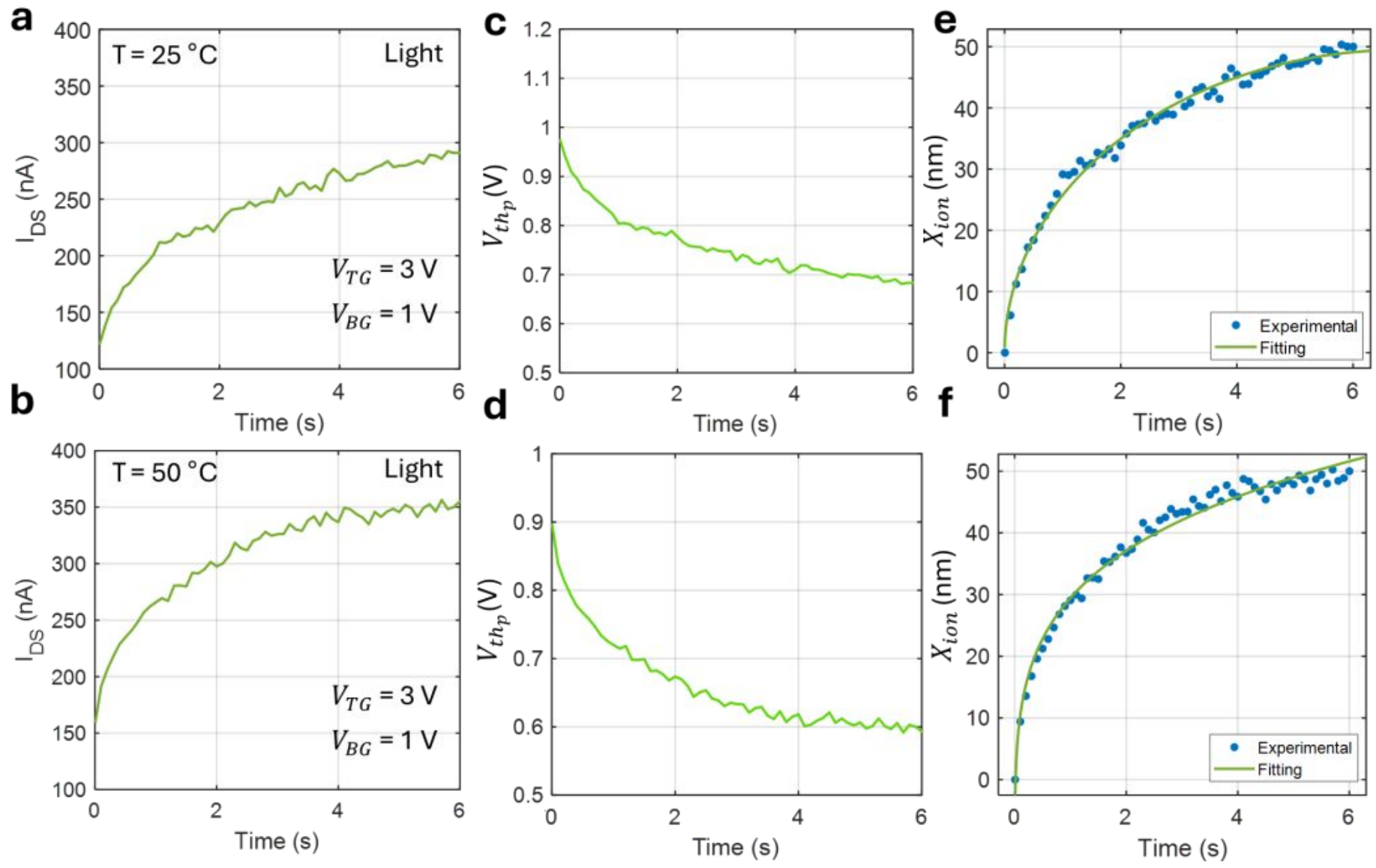

Supplementary Information 17. Virtual-Source Model for assessing V_{th} shift in AIPS-gated 2D FETs under light. *(a-b) source-to-drain current (I_{DS}) of a MoS_2 FET as a function of time, for a constant top-gate voltage (V_{TG}) of 3 V and V_{DS} = 1 V, measured across different temperatures. Over time, the migration of Ag^+ ions within AIPS gradually shifts V_{th_p}, leading to a continuous and monotonic rise in I_{DS}. (c-d) Extracted V_{th_p} evolution over time exhibits a steady, monotonic decrease. Notably, the change in V_{th_p} is accelerated at higher temperatures, attributed to the enhanced ionic mobility. (e-f) Evolution of the Ag^+ ion charge distribution centroid, X_{ion}, over time, presented alongside a theoretical fit.*

Supplementary Information 18

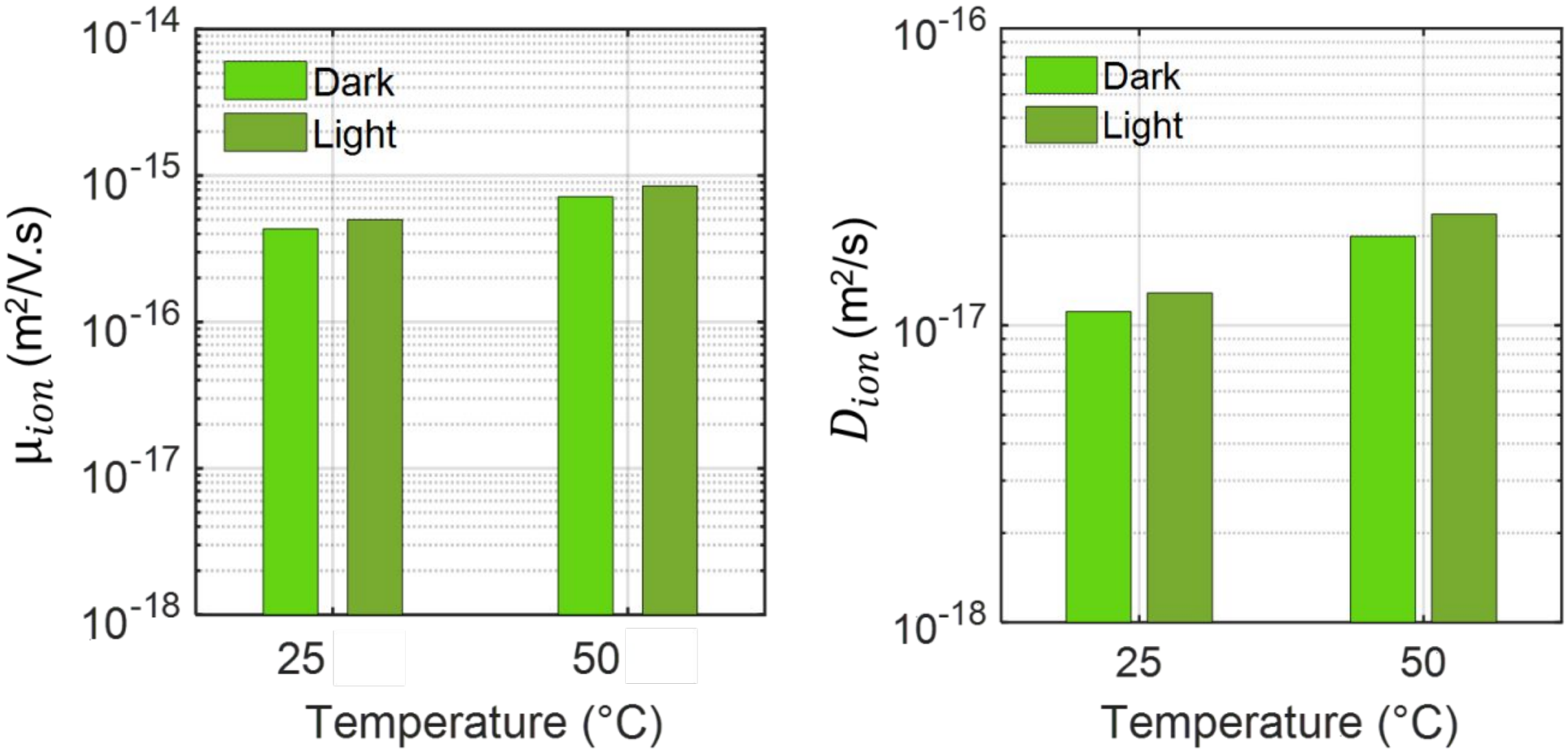

Supplementary Information 18. Ionic mobility (μ_{ion}) and diffusivity (D_{ion}) in AIPS. *Apparent ionic mobility (μ_{ion}) and effective diffusivity (D_{ion}) for Ag^+ ions in AIPS, extracted for T=25 °C and 50 °C, under dark and illumination conditions, respectively. These results show a clear increase with rising temperature, consistent with thermally activated ion transport. Interestingly, upon illumination, photoexcited carriers interact with the local defect landscape, enhancing the drift of Ag^+ ions within the lattice. This photo assisted ionic motion accelerates the redistribution of Ag^+ ions at the AIPS/MoS_2 interface, leading to a faster and more pronounced modulation of the channel conductance.*

Supplementary Information 19

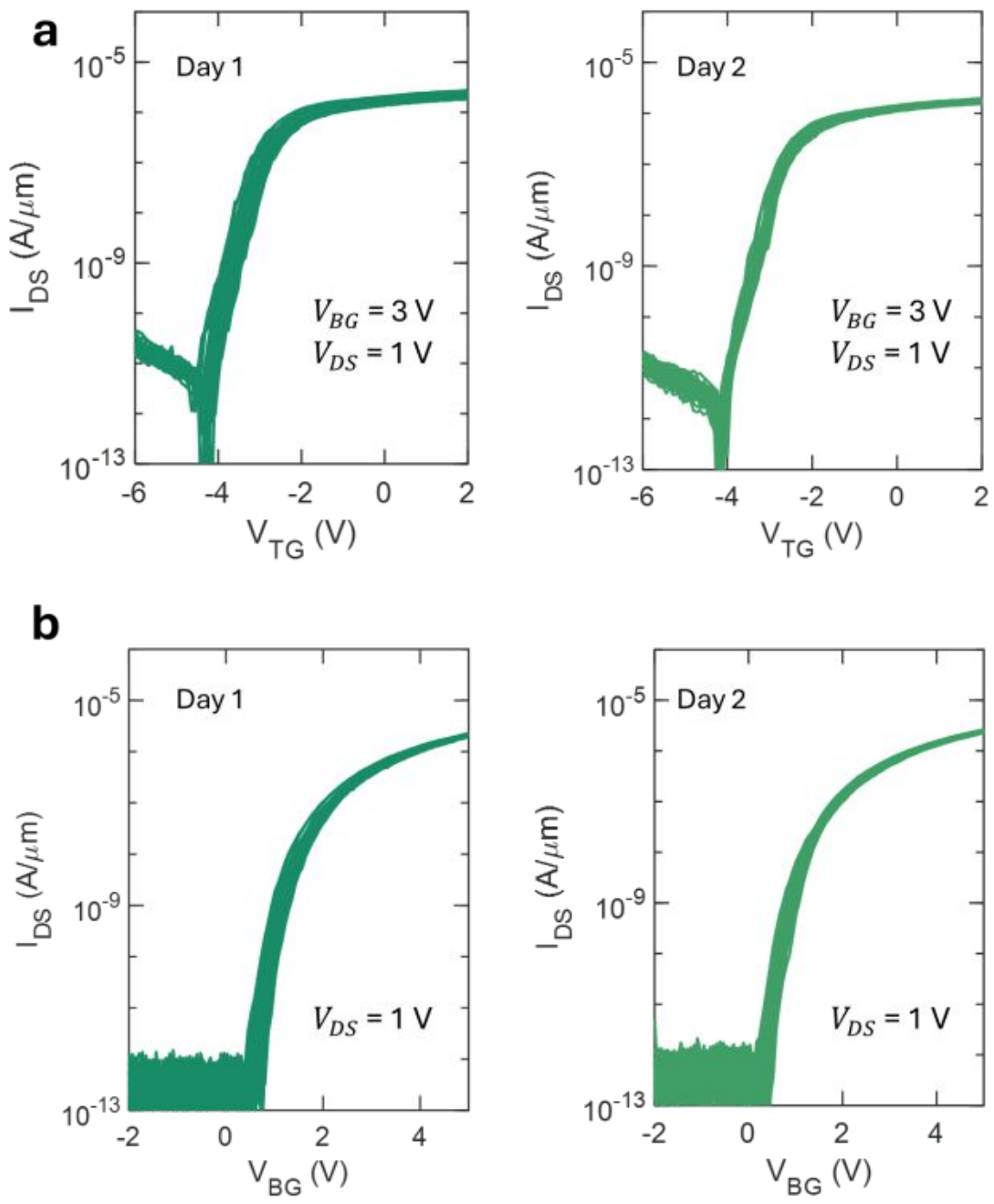

Supplementary Information 19. Long-term operational stability of top and back-gated MoS_2 FETs. *a) Top-gate transfer characteristics of an AIPS-gated MoS_2 FET was measured by sweeping V_{TG} from -6 V to 2 V at a constant V_{BG} =3 V and V_{DS} =1 V, over two consecutive days for multiple cycles. The results reveal minimal variations in the top-gate threshold voltage, confirming excellent operational stability. b) Back-gate transfer characteristics were measured over two consecutive days for multiple cycles, without any encapsulation layer displaying negligible variation in the threshold voltage.*

Supplementary Information 20

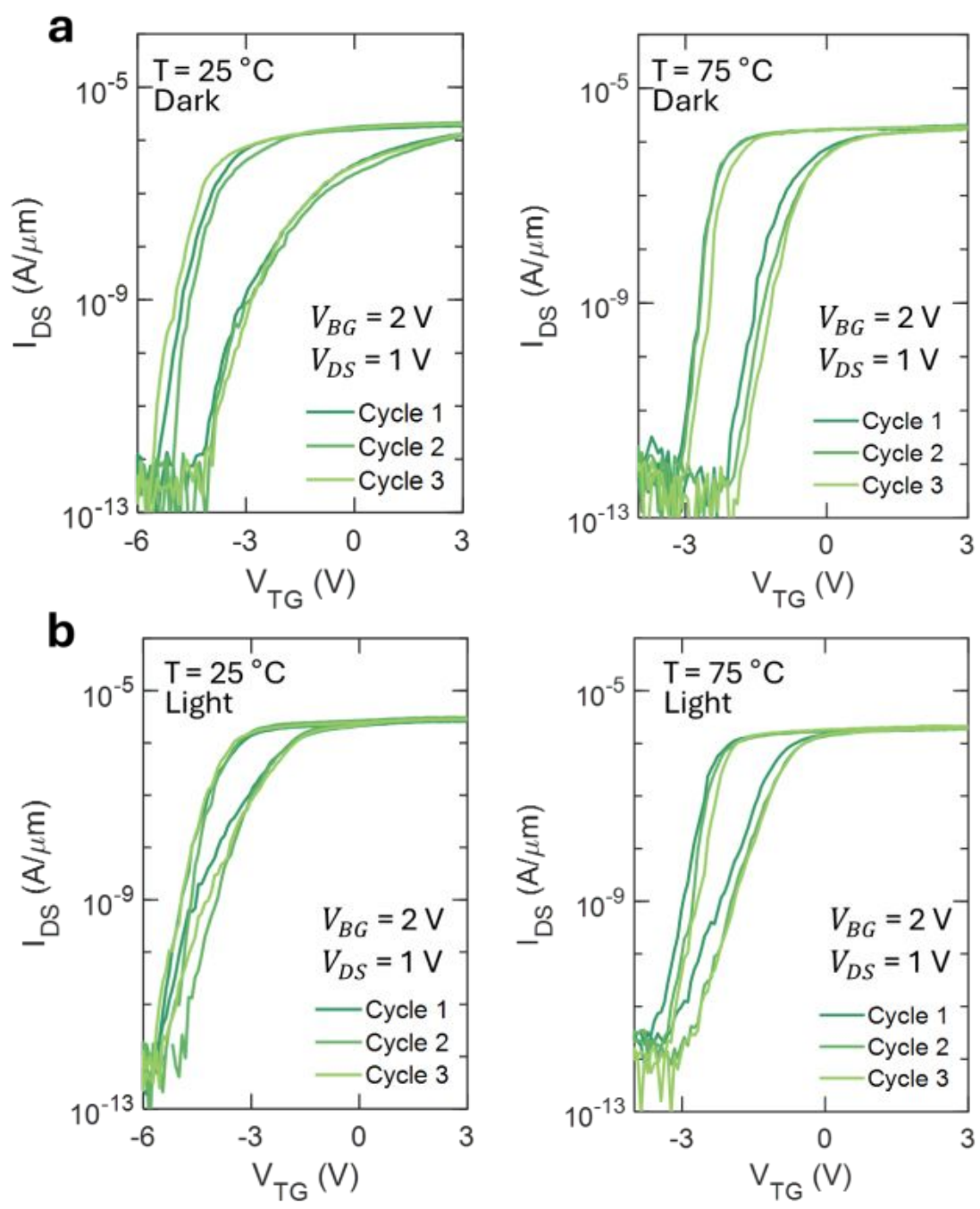

Supplementary Information 20. Thermal cycling in top-gated MoS_2 FET with AIPS dielectric. *Dual-sweep top-gate transfer characteristic of an AIPS-gated MoS_2 FET obtained by sweeping the V_{TG} across multiple thermal cycles, with temperatures ranging from 75 °C to 25 °C, under a) dark and b) illumination. These results show minimal variation across successive cycles, with the device consistently retracing the same transfer characteristics under thermal stress. This consistent behavior confirms that Ag^+ migration within the dielectric is fully reversible under thermal excitation and relaxation, and that no cumulative ionic displacement or defect generation occurs during repeated operation.*

Supplementary Information 21

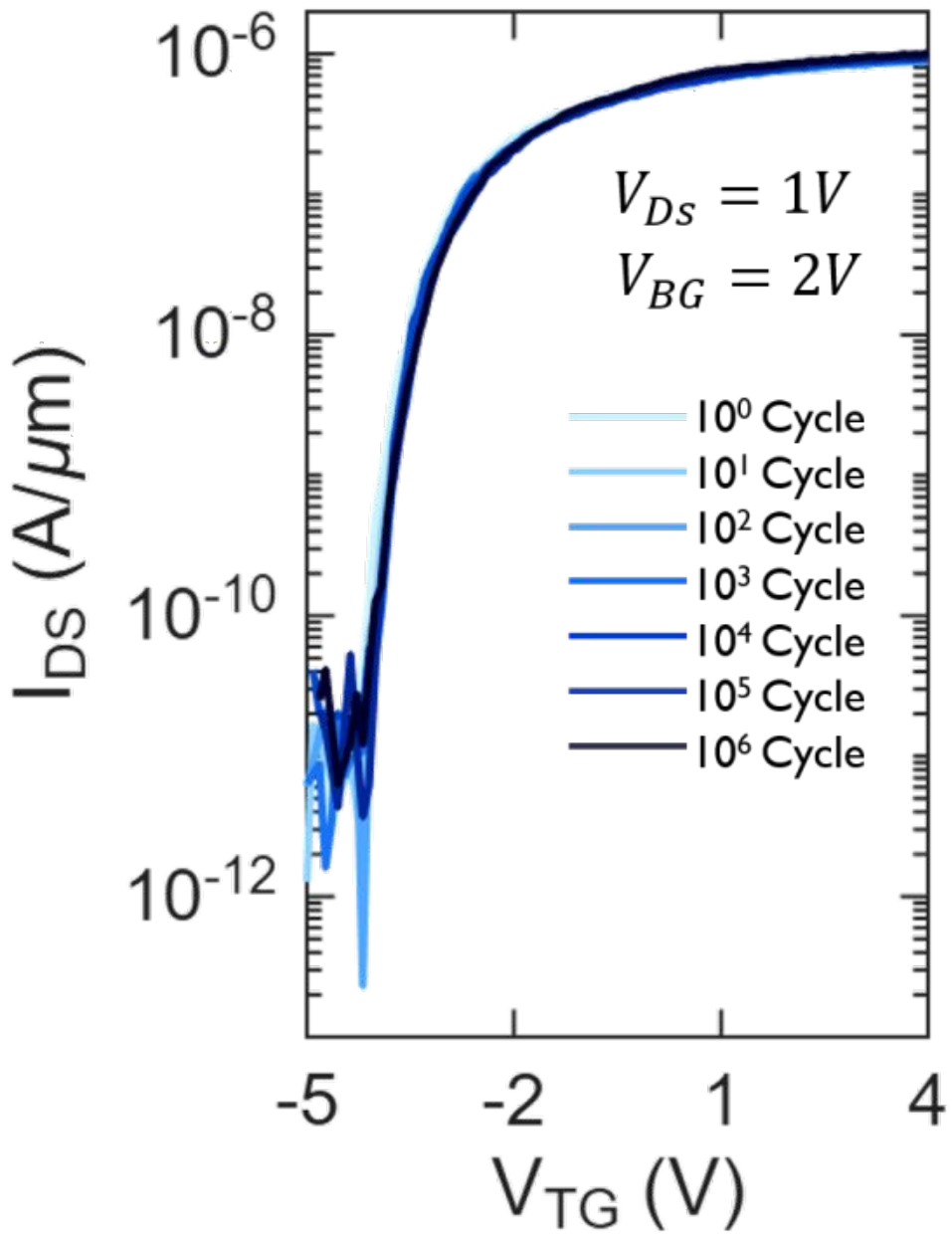

Supplementary Information 21. Electrical endurance of AIPS-gated MoS_2 FETs. *Top-gate transfer characteristics measured after repeated electrical cycling from 10^0 to 10^6 cycles at* $V_{DS} = 1$ V *and* $V_{BG} = 2$ V. *The nearly identical transfer curves over six orders of magnitude in cycling demonstrate excellent device endurance and operational stability, with negligible degradation in threshold voltage, subthreshold characteristics, or on-state current.*

Supplementary Information 22

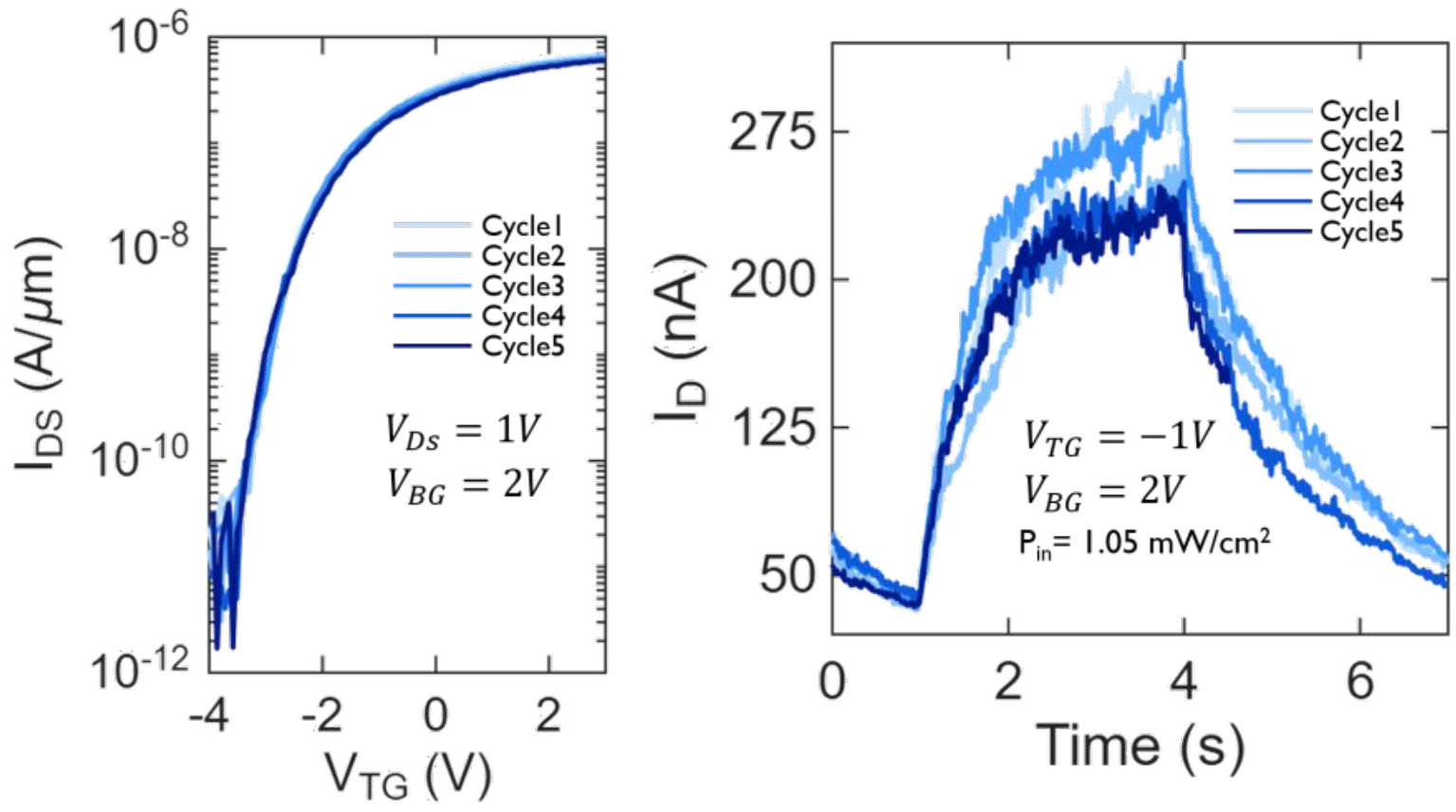

Supplementary Information 22. Cycle-to-cycle reproducibility of photo-responses in AIPS-gated 2D FETs. *Top-gate transfer characteristics measured over five consecutive cycles at* $V_{DS} = 1$ V *and* $V_{BG} = 2$ V. *The excellent overlap of the transfer curves demonstrates stable transistor operation with negligible cycle-to-cycle variation. Time-resolved photo-response measured over five consecutive illumination cycles at* $V_{TG} = -1$ V, $V_{BG} = 2$ V, *and* $P_{in} = 1.05\ mW\ cm^{-2}$. *The reproducible photocurrent evolution during both illumination and relaxation confirms the stability of the opto-ionic response and the repeatability of the photo-induced conductance modulation in AIPS-gated 2D FETs.*

Supplementary Information 23

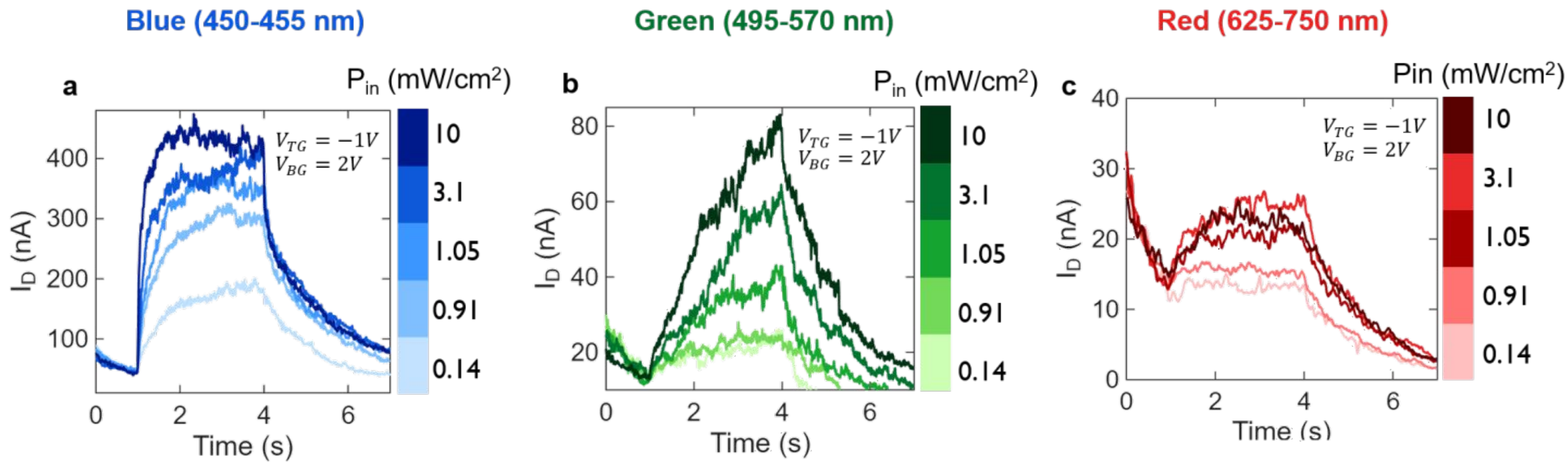

Supplementary Information 23. Wavelength-dependent photo-response of AIPS-gated 2D FETs. *Time-resolved drain current measured under blue (450–455 nm), green (495–570 nm), and red (625–750 nm) LED illumination at different incident power densities. The photo-response exhibits a pronounced wavelength dependence, with the largest conductance modulation observed under blue illumination, followed by green illumination, while the response is strongly suppressed under red illumination.*
